# Clearance pathways of near-infrared-II contrast agents

**DOI:** 10.7150/thno.79209

**Published:** 2022-11-14

**Authors:** Sha Yang, Na Li, Hao Xiao, Gui-long Wu, Fen Liu, Pan Qi, Li Tang, Xiaofeng Tan, Qinglai Yang

**Affiliations:** 1 Center for Molecular Imaging Probe, Hunan Province Key Laboratory of Tumor Cellular and Molecular Pathology, Cancer Research Institute, Hengyang Medical School, University of South China, Hengyang, Hunan 421001, China.; 2 Tumor Pathology Research group & Department of Pathology, Institute of Basic Disease Sciences & Department of Pathology, Xiangnan University, Chenzhou, Hunan 423099, China.; 3 Key Laboratory of Tropical Medicinal Plant Chemistry of Ministry of Education, College of Chemistry and Chemical Engineering, Hainan Normal University, Haikou, Hainan 571158, China.

**Keywords:** near-infrared-II, *in vivo* imaging, hepatobiliary clearance, renal clearance, biocompatibility

## Abstract

Near-infrared-II (NIR-II) bioimaging gradually becomes a vital visualization modality in the real-time investigation for fundamental biological research and clinical applications. The favorable NIR-II contrast agents are vital in NIR-II imaging technology for clinical translation, which demands good optical properties and biocompatibility. Nevertheless, most NIR-II contrast agents cannot be applied to clinical translation due to the acute or chronic toxicity caused by organ retention *in vivo* imaging. Therefore, it is critical to understand the pharmacokinetic properties and optimize the clearance pathways of NIR-II contrast agents *in vivo* to minimize toxicity by decreasing organ retention. In this review, the clearance mechanisms of biomaterials, including renal clearance, hepatobiliary clearance, and mononuclear phagocytic system (MPS) clearance, are synthetically discussed. The clearance pathways of NIR-II contrast agents (classified as inorganic, organic, and other complex materials) are highlighted. Successively analyzing each contrast agent barrier, this review guides further development of the clearable and biocompatible NIR-II contrast agents.

## 1. Introduction

NIR-II bioimaging has attracted growing attention in biomedical fields, especially as a method of non-invasive real-time monitoring *in vivo*, showing great prospects for clinical transformation 1-5. Owing to suppressed photon scattering, diminished absorption, and lower tissue autofluorescence in the NIR-II region (1000-1700 nm), the NIR-II window manifests superiority of high spatiotemporal resolution with increasing tissue penetration depths 6-10, which provide more significant and potential applications for *in vivo* detection to explore physiological processes 11-14. Encouraged by the application potential of NIR-II bioimaging, a variety of NIR-II contrast agents have been developed 15-22. According to the chemical composition of luminous cores, NIR-II contrast agents generally be classified into inorganic-based, organic molecule-based, and organic nanoparticle-based materials. Inorganic-based contrast agents consist of an inorganic optical core (*e.g.,* single-walled nanotubes (SWNTs), quantum dots (QDs), rare earth doped-nanoparticles (RENPs), carbon-based nanoparticles) and a water-soluble shell (*e.g.,* polyethylene glycol (PEG), aptamer, cyclodextrin) 23-29. In comparison, organic molecular contrast agents are comprised of an organic optical moiety (hydrophobic conjugated skeleton) and a water-soluble moiety (*e.g.,* PEG, cyclodextrin, and sulfonate ion) 30-34. In addition, organic nanoparticle-based contrast agents are comprised of a molecular dye (*e.g.,* polymethine, D-A-D structured contrast agents) encapsulated in an amphiphilic matrix (*e.g.,* DSPE-PEG, PS-PEG, protein) 35-37.

The existing NIR-II contrast agents have powerful advantages in the field of *in vivo* and *in vitro* detection*.* The point-of-care testing based on the NIR-II contrast agents has been extensively applied for *in vitro* diagnostics 37. However, there is no report concerning clinically approved NIR-II contrast agents for *in vivo* imaging. Up to now, only some clinically approved NIR contrast agents, such as indocyanine green (ICG) and methylene blue (MB), could exhibit a tail fluorescence in the NIR-II region 38-41. Therefore, developing clinically approved contrast agents with prominent peaks in the NIR-II window is still essential. However, the favorable NIR-II contrast agents for clinical translation demand superior optical properties and biological safety 42-45.

Given this, the pharmacokinetics of NIR-II contrast agents are crucial awfully. In brief, there are three main pharmacokinetic pathways for the degradation and clearance of bioimaging materials, including renal, hepatobiliary, and MPS clearance pathways 46-49. Generally, the optical core of NIR-II contrast agents is required to maintain a sustained and steady imaging signal, thus hard to be degraded within a short period in the body. The unpredictable long-term toxicity caused by the retention of contrast agents in organs is a major obstacle to achieving *in vivo* NIR-II imaging for short-term clinical transformation 50, 51. According to Food and Drug Administration (FDA) requirements, all the injected imaging diagnostic agents are cleared from the body within a reasonable time 52-54. Therefore, it is indispensable to study the fate of NIR-II contrast agents *in vivo* and develop clearable NIR-II contrast agents with reduced biological toxicity, further promoting the clinical translation of NIR-II technology 55-57.

An important principle in designing a drug delivery system is overcoming biological barriers and facilitating drugs arriving at the lesion sites. Moreover, Other drugs dissociated from the lesion sites should be eliminated by the biological clearances to avoid adverse effects 58, 59. Normally, NIR-II contrast agents have molecule and nanoparticle forms and could act as drug delivery systems for the maximum aggregation in the target sites 60-62. The contrast agents that fail to reach the lesion sites can be cleared from the body by hepatobiliary, MPS, or renal clearance pathways 63-65, avoiding the false positive signals and unpredictable toxicity caused by organ retention 51, 66, 67. Considering the clearance principle of biomaterials, the chemical composition, size, shape, and surface charge of biomaterials have a crucial impact on biological distribution and clearance 68-70. Large-sized materials would be mainly assimilated by MPS because of the plasma protein adsorption on the materials, triggering sequestration by MPS. If the large-sized materials enter the liver, they will be internalized by hepatocytes, followed by the clearance through the bile and feces within days to months, resulting in unpredictable chronic toxicity 71, 72. Several pieces of literature demonstrated that when contrast agents interact with biological tissues, the tissues will induce oxidative stress by producing superactive free radicals, leading to genotoxicity, mitochondrial damage, lysosomal dysfunction, and cell necrosis or apoptosis eventually. The toxicity induced by contrast agents largely depends on their physicochemical characteristics (*e.g.,* size, surface chemistry, and morphology), dosing regimen, route of exposure, and immunogenicity. Therefore, the rational design of contrast agents with optimal physicochemical properties can avoid unnecessary biotoxicity 73. For example, small-sized materials are mainly cleared through the kidney and cleared quickly from the body within minutes to hours of post-injection (p.i.), which is beneficial for reducing long-term organ toxicity 74, 75.

**Figure 1:**
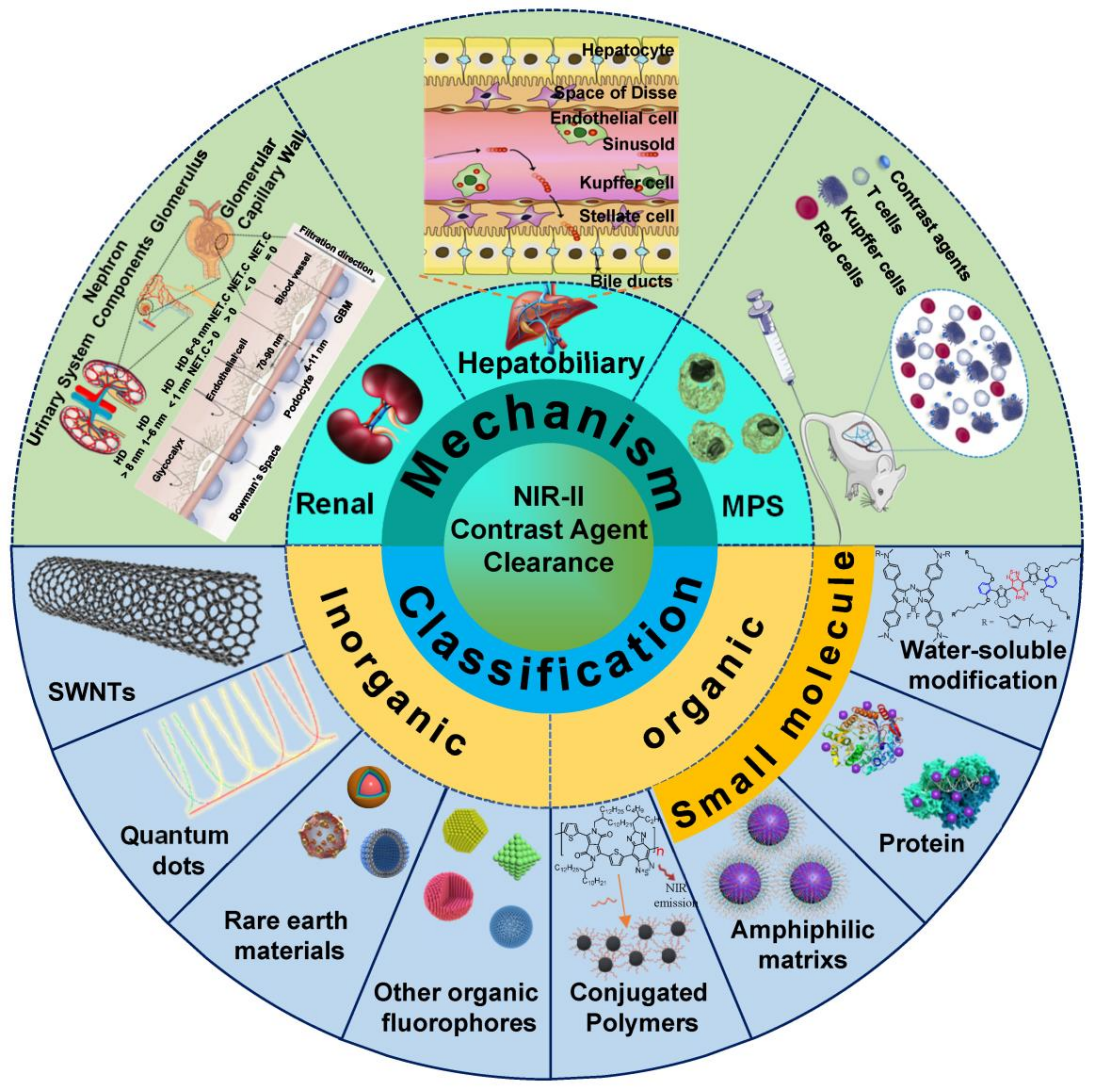
Clearance pathways of NIR-II contrast agents.

In terms of quick excretion, the renal clearance pathway can effectively alleviate safety concerns of exogenous contrast agents, which is superior to hepatobiliary and MPS clearance pathways. Therefore, NIR-II contrast agents must be redesigned to achieve renal clearance for improving biocompatibility, which is helpful to accelerate the NIR-II bioimaging technology for clinical transformation. In this review, we first synthetically discuss the clearance mechanisms of renal, hepatobiliary, and MPS clearance pathways. The clearance pathways of NIR-II contrast agents based on inorganic, organic, and other complex materials are classified and gathered. Successively analyzing each contrast agent clearance mechanism based on structural characteristics (Figure 1), this review provides rational guidance for further development of clearable and biocompatible NIR-II contrast agents.

## 2. Clearance mechanisms for contrast agents

The clearance mechanism is a necessary biological process to prevent damage and toxicity from the body by eliminating contrast agents 76, 77. Once the contrast agents (*e.g.,* drugs, microorganisms, and other granular materials) enter into blood circulation, they are cleared through three clearance pathways, including MPS, hepatobiliary, and renal clearance in the metabolic process 78. External substances will interact with serum proteins in the blood circulation to form the protein corona, called opsonin. MPS refers to binding this opsonized stuff by complement factors, fibrinogen, and immunoglobulins during the circulation process 79. The contrast agents are phagocytosed and degraded to produce degradants in cells, whereafter they can be absorbed for biological recycling or drawn into the blood circulation system for clearance. If the contrast agents are non-degradable in these processes, they will remain in cells (such as Kupffer cells) and be sequestered in the spleen and liver for months to years, which would cause the concerns of chronic toxicity. During the process of hepatobiliary clearance, hepatocytes in the liver also remove nanoparticles through endocytosis, then enzymatically broken down and cleared by the biliary system into the bile *via* the fecal clearance. The entire process could be required for hours or even days 77, 80. By comparison, as for the kidneys, some invading substances are rapidly filtrated by the glomerular filtration membrane, transported by the renal tubules, then discharged into the bladder, and cleared through urine eventually. The kidney clearance process can be shortened to minutes or hours 51, 81, 82. Therefore, compared to the clearance pathways of MPS and hepatobiliary, renal route is the most effective clearance pathway.

**Figure 2:**
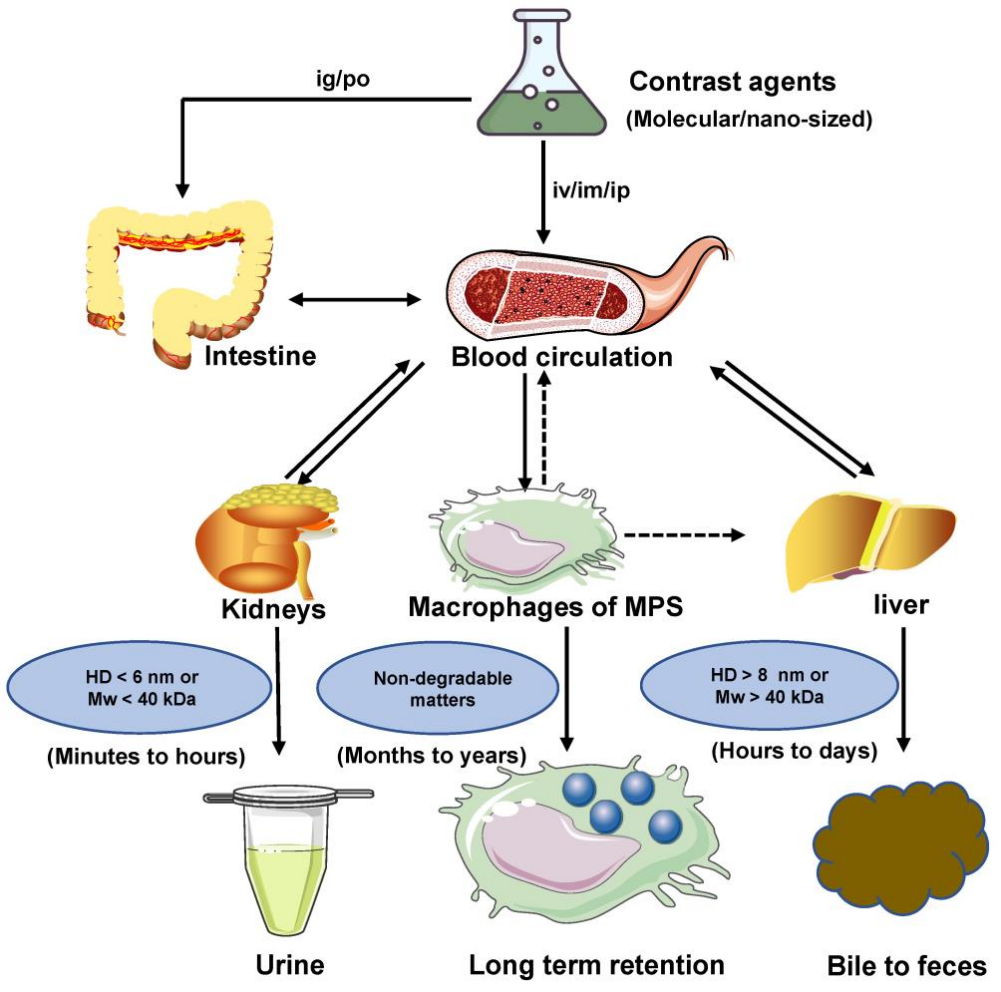
** Clearance pathways of contrast agents.** Contrast agents are cleared through the kidney, liver, and MPS pathways. Solid arrows represent the most probable interaction, and dashed arrows mean possible interaction. If the contrast agents with HD < 6 nm or MW < 40 kDa, they can be cleared by renal clearance within minutes to hours after administration. If the contrast agents with HD >8 nm or MW > 40 kDa, they can be cleared by hepatobiliary clearance within hours to days after administration. Non-degradable or larger-sized matters are more likely to be internalized and retained by MPS. If MPS degrades the contrast agents, their decomposer may avoid sequestration and return to the blood circulation for eventual renal or hepatobiliary clearance. Notably, MPS includes the Kupffer cells in the liver.

The clearance routes for contrast agents are determined by several comprehensive factors, including composition, size, shape, charge, and surface modification of contrast agents. The contrast agents with a small hydrodynamic diameter (HD) (< 6 nm) or a small molecule weight (MW) (< 40 kDa) can pass through the glomerular capillary wall for the renal clearance pathway 83. In comparison, agents with large size or weight (>8 nm or 40 kDa ) are frequently sequestered from the blood circulation and eliminated by MPS and hepatobiliary clearance pathways. Notably, some large-size or high molecule weight of agents can also be removed through kidneys after phagocyted degradation in a long period of circulation *in vivo* 32, 84. In blood circulation, the contrast agents with a neutral or slightly negative surface chargeare benefical. In contrast, the contrast agents with positive charges contribute to the uptake of cells in the phagocytic system leading to a short half-life 85. The contrast agents coated with the hydrophilic polymer would reduce the uptake rate of MPS 49. For example, polyethylene glycol (PEG) is a hydrophilic polymer with good water solubility, high solution fluidity, non-toxicity, and rapid clearance* in vivo* 86, 87. In terms of the shapes, the rod shape is beneficial to avoid serum protein accumulation, leading to a decrease in the uptake rate of MPS 49, 69. Typically, the diagnostic and therapeutic agents entering the circulatory system can be biologically utilized by degradation and cleared through feces or urine. Otherwise, bioaccumulation leads to unpredictable chronic toxicity. The three clearance pathways of MPS, hepatobiliary and renal, are directly or indirectly associated with blood circulation and phagocyte degradation and work together to complete the task of contrast agents clearance (Figure 2) 80, 88.

### 2.1. Renal clearance mechanism

**Figure 3:**
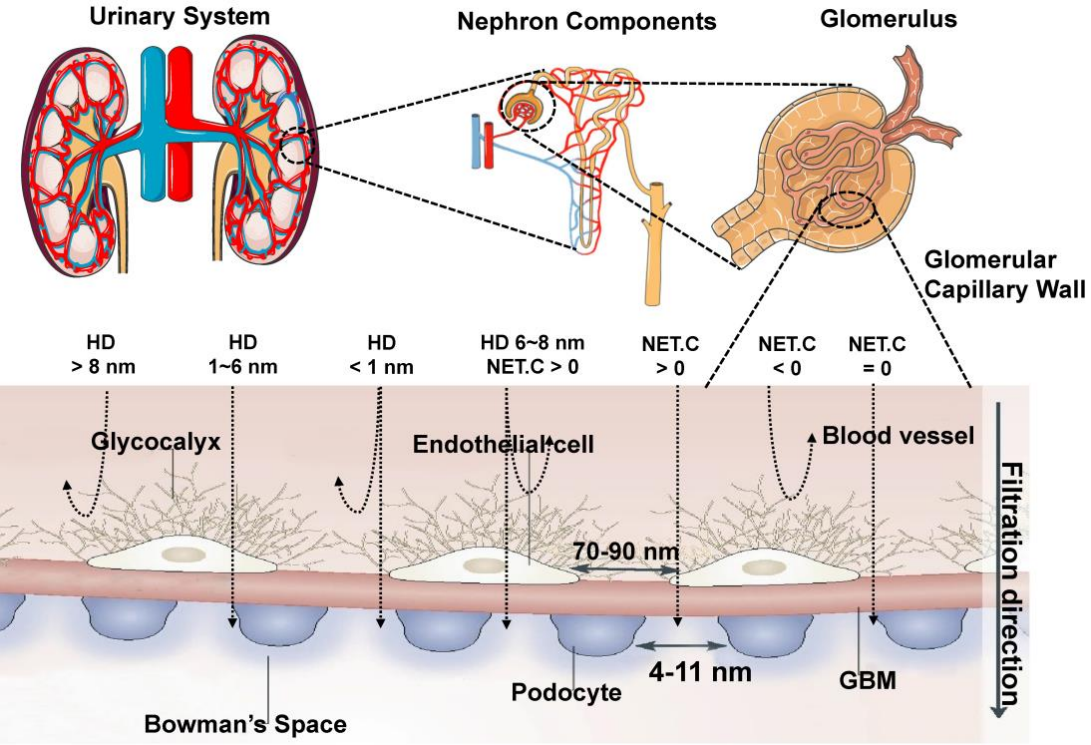
Transport of contrast agents with different sizes and surface charges in the urinary system (reproduced from ref. 82 with permission from Springer Nature and ref. 95 with permission from Elsevier).

The glomerular filtration membrane (GFM) is made up of an endothelial glycocalyx, endothelial cells, the glomerular basement membrane (GBM), and podocytes, which is supported by mesangial cells (or mesangium) in the mesangial matrix 89, 90, performing kidney filtration. Mesangial cells located between glomerular capillaries 91 are also played a critical role in regulating glomerular filtration. The glycosylated endothelial calyx consists of glycosaminoglycans (*e.g.,* heparan sulfate, chondroitin sulfate, and hyaluronic acid) and related proteoglycansm, which play a vital role in renal filtration, such as preventing proteins with negative charge leakage 92. The endothelial layer comprises monolayer endothelial cells with 70-90 nm perforation, and the GBM comprises collagen type II, laminin, and proteoglycan. Collagen, proteoglycans (mainly heparin sulfate), and laminin form a network with pores in the range of 2-8 nm. On the other side of the GBM, the podocytes are located and face the Baumann space. The podocyte layer possesses a pore diameter of 4~11 nm and is covered with a sugar calyx of 200 nm thick 82. In conclusion, the renal filtration barrier comprises mechanical and charge barriers. Studies have shown that the renal clearance efficiency of contrast agents mainly depends on their sizes, shapes, protein binding affinities, macrophage uptake, and surface modifications 66, 93. Theoretically, only small-sized matters less than the renal filtration threshold of 6 nm can be filtered. Whereas large-sized materials larger than 8 nm cannot filter through the renal system, resulting in increased aggregation of contrast agents in MPS. The surface charge of contrast agents affects the renal clearance rate 82. The efficiency of renal clearance increased with the decrease of zeta potential, indicating that the contrast agents with a strong negative charge can be removed faster than those with a weak negative charge or with a positive charge, which seems to contradict the general understanding of the charge-dependent glomerular filtration. In addition, under the same net surface charge, the zwitterionic surface ligands may be conducive to faster removal than the single-charged surface ligands 94. Because the glomerular endothelial wall in renal systems is closely related to the size and surface charge, the neutral-charge surface modification is also critical for contrast agents with an HD of 6-8 nm for renal clearance. Agents with a positive charge are more different for glomerular filtration than the neutral ones, and the glomerular filtration rate is higher for negative charge matters than the neutral ones (Figure 3) 95. For nano-sized matters smaller than 6 nm, the size is a major factor in renal clearance other than the surface charge, while for the size larger than 6 nm, the surface charge is a dominant factor in renal clearance 82. Moreover, the effective renal clearance nanostructures with a diameter of 1-2 nm can interact with the endothelial glycocalyx with low removal efficiency 95. Except for spherical ones, nano-sized matters are usually designed to be rods, tubes, and sheets. For example, flaky graphene oxide (GO) was observed in urine with rapid renal clearance 96, indicating that non-spherical and nano-sized matters play an important role in the renal filtration. Besides, surface modification also played a role in renal clearance. For example, zwitterionization and PEGylation are two extensively used surface modulation strategies for renal-clearable nanoparticles to impart them with a neutral surface charge property 97. When contrast agents enter the blood circulation, low or no uptake of macrophages is more beneficial to entering the kidney, thus speeding up the clearance. Therefore, NIR-II contrast agents are benefit for eliminating through the kidney should have properties with a small size (the renal filtration threshold of MW: ~ 40 kDa or HD: ~ 6 nm), electrical neutralization, rapid dissociation from serum protein, and low macrophage uptake, providing significant guidance for the rational design of renal-clearable NIR-II contrast agents.

### 2.2. Hepatobiliary clearance mechanism

**Figure 4:**
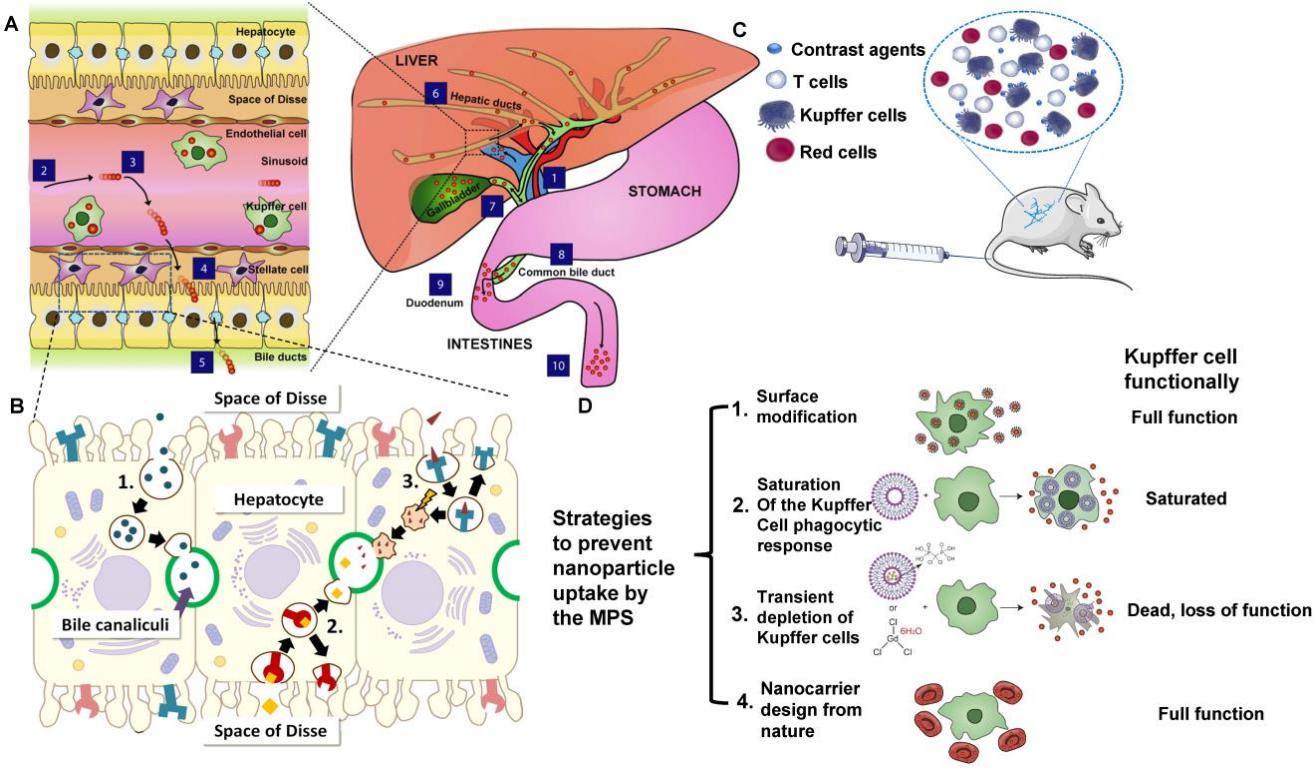
** Transport of contrast agents in hepatobiliary system and MPS.** (**A**) Schematic illustration of the hepatobiliary processing and clearance of contrast agents: (1) enter into the liver *via* the portal vein, (2) matters across the hepatic sinusoid, (3) be likely to accumulate in Kupffer cells, (4) probably leak into the space of Disse and be endocytosed by hepatocytes, (5) transcytose *via* the hepatocytes and enter into the bile duct *via* bile canaliculi, (6) go through the hepatic ducts, (7) Contrast agents may first collect in the gall bladder, (8) Contrast agents may enter into the common bile duct, (9) Contrast agents may be cleared into the duodenum of the small intestines *via* the sphincter of Oddi, (10) eventually cross the entire gastrointestinal tract and are cleared in feces (reproduced from ref. 80 with permission from Elsevier). (**B**) Different biliary elimination processes of colloids by hepatocytes: (1) fluid-phase endocytosis (pinocytosis), (2) direct receptor-mediated transcytosis, and (3) receptor-mediated transcytosis followed by passage through lysosomes. The shock symbol denotes acidic and oxidative conditions (reproduced from ref. 99 with permission from Elsevier). (**C**) Contrast agents that are not toxic to the cell function or mobility can be effectively uptaken by circulating blood-borne monocyte-macrophages and then transported to the areas of the lymphoid and MPS (for example, liver and spleen). (**D**) Scheme of strategies to prevent contrast agents uptake by MPS, especially Kupffer cells (reproduced from ref. 80 with permission from Elsevier).

The hepatobiliary system is one of the primary clearance ways of contrast agents that fail to be cleared by the kidney. The liver has the vital functions of catabolism and bile clearance of blood circulating particles by eliminating contrast agents with phagocytosis. Modified with specific surface ligands after intravenous administration, molecular or nano-size contrast agents could be recognized *via* cellular targets or their receptors on a specific type of liver cells, including hepatocyte, Kupffer cell, hepatic stellate cells (HSC), and endothelial cells. Especially, hepatocytes, as the vital functional cells in the liver (occupy 70-85% of the liver cell population) play a critical role in endocrine, metabolic, and secretory functions 98. In the process of hepatobiliary clearance, the contrast agents in hepatic sinusoids enter the space of Disse through the fenestra of vascular endothelial cells. The contrast agents are recognized and internalized by hepatocytes by receptor-mediated endocytosis/clathrin-mediated endocytosis or other pathways (Figure 4A) 80. Soon after, those agents are broken down with enzymes in the hepatocytes and discharged into the capillary bile duct on the other side of the hepatocytes (Figure 4B) 99. Finally, they are cleared into the feces through the biliary system 80. Notably, all the physical or chemical catabolism in the hepatobiliary clearance process is more complicated than renal clearance.

Although the phagocytosis of hepatocytes is much lower than that of Kupffer cells, the liver comprises a large number of hepatocytes, which can absorb the large-sized (HD > 20 nm) 100, hydrophobic, positively charged molecular or nano-sized matters 101, and remove them from the body through the hepatobiliary pathway 80. It should be noted that the absorption of contrast agents from the blood to the liver is relatively fast. Nevertheless, the liver processing and biliary clearance for these contrast agents are relatively slow, which prolongs the retention time in the liver parenchyma with potential toxicity 75.

### 2.3. MPS clearance mechanism

The clearance of MPS refers to removing contrast agents by phagocytes in the blood and tissues. Contrast agents with high cationic and anionic surface charges can adsorb many serum proteins to form a protein “corona” that aggregates* in vitro*, showing a strong interaction with macrophages 102.

MPS comprises circulating monocytes, spleen red pulp, marginal zone macrophages, Kupffer cells, bone marrow macrophages, and sinusoid endothelial cells 103. The large-sized contrast agents can be absorbed and cleared by MPS after the interactions with proteins 85, such as the adsorption of serum protein, especially the opsonization of complement, fibrinogen, and immunoglobulin, thus could be recognized and cleared through MPS (Figure 4C) 104-106. When endocytosed by phagocytes, contrast agents will be degraded or remain in the spleen and liver for more than 6 months 107-109. Once the phagocytes are filled with contrast agents, they will be re-uptaken by other phagocytes in the same organ, causing accumulation in the Kupffer cells of the liver 108. Herein, we take Kupffer cells as an example to briefly illustrate the clearance mechanism of contrast agents in MPS. In brief, circulating mononuclear cells in the blood can adhere to liver tissues and be polarized into the Kupffer cells with highly differentiated surface receptors. Those Kupffer cells, accounting for 80-90% of macrophages in the body 110, are located in the liver sinusoid that engulfs and removes pathogens and other external substances from the blood *via* the macrophage phagocytosis, reticulate-mediated or caveolin-mediated endocytosis 111. Contrast agents that cannot be decomposed by the internalization of those cells will remain in the body for a long time 107.

Some strategies were put forward to prevent MPS from uptaking external agents (Figure 4D) 80, 112. For instance, some molecular or nano-sized matters modified by PEG can prevent or reduce serum protein opsonization and increase the serum half-life of nanoparticles 75. which is not easy to be identified by MPS 113. The large-sized molecular or contrast agents tend to be cleared through hepatobiliary or MPS clearance pathways, and the biotransformation in hepatocytes and macrophages might have potential biotoxicity 80.

## 3. Clearance pathways of NIR-II contrast agents

The clearance pathways of NIR-II contrast agents, including inorganic, organic, and other complex materials, are listed in **Tables 1** and **2**. Furthermore, systemically analyzing each contrast agent clearance mechanism based on the structural characteristics provides rational guidance for further developing the clearable and biocompatible NIR-II contrast agents.

### 3.1. Clearance pathways of inorganic NIR-II contrast agents

#### 3.1.1 SWNTs

**Figure 5:**
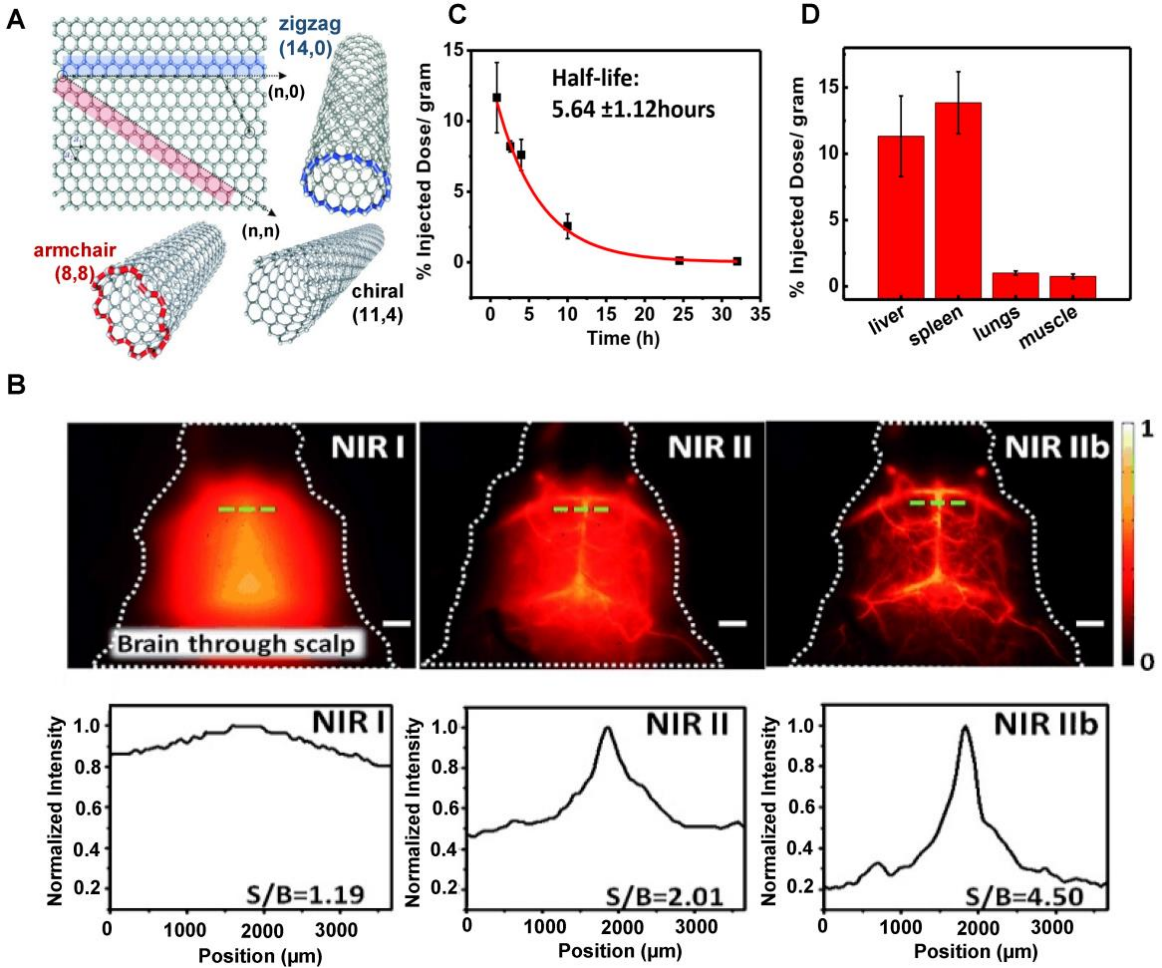
** Clearance of SWNTs.** (**A**) Structure of single-walled carbon nanotubes (reproduced from ref. 142 with permission from Wiley-VCH Verlag GmbH. (**B**) SWNTs fluorescence images of the cerebrovasculature of the mice without craniotomy in the NIR-I, NIR-II, and NIR-IIb regions and with the corresponding signal-to-background (SBR) analysis. (**C**) The half-life of SWNTs blood circulation. (**D**) The semiconducting LV SWNTs accumulation in the liver (11% ID/gram) and spleen (14% ID/gram) of MPS (reproduced from ref. 115 with permission from Wiley-VCH Verlag GmbH).

Since Iijima discovered carbon nanotubes in the process of preparing C_60_ by the arc discharge in 1991 for the first time, carbon nanotubes have attracted close attention in the research field of nanotechnology all over the world owing to their unique structure and excellent electrical as well as mechanical and optical properties 141. The interrelated carbon atoms between layers completely roll up carbon nanotube structures. The diameter is about 1~150 nm, and the length can reach the centimeter level 142. According to the number of tube wall layers, carbon nanotubes can be divided into the following two categories: i) single-walled nanotubes (SWNTs) are cylindrical seamless tubes coiled from the single-layer graphite with diameters between 0.4~3 nm (Figure 5A) 142; ii) multi-walled nanotubes (MWNTs) are comprised of several coaxial single-walled carbon nanotube sleeves separated by ~ 0.34 nm with diameters between 14~150 nm. The *in vivo* clearance of SWNTs largely depends on surface functionalization, such as the length and structure of functional molecules 143-145.

In 2002, Smalley group firstly discovered that SWCNTs could emit fluorescence in the NIR band 146. After that, the applications of SWCNTs in the NIR optical imaging are well recognized. SWCNTs show strong fluorescence in the NIR-II wavelength range of 1000-1700 nm with almost no toxicity and photobleaching and are considered an ideal molecular imaging probe. SWCNTs modified with PEG could result in long blood circulation, low uptake by MPS, and relatively rapid clearance. In 2009, Dai group applied SWNTs to image animals for the first time 147. A layer of the phospholipid polyethylene glycol (PL-PEG) molecules was modified on the surface of SWNTs to prepare a new type of PL-PEG functionalized SWNTs. *In vivo* imaging results showed that PL-PEG had a strong NIR-II fluorescence signal, good tissue penetration ability, and clear images in the vascular system under the skin. In addition, Dai group further studied the possibility of applying polyethylene glycol-conjugated phospholipid (DSPE-mPEG) functionalized SWCNTs to deep tissue anatomical imaging based on the above experiments 114. SWNTs modified by DSPE-mPEG could realize deep tissue imaging and could be cleared through MPS. On the first day of the intravenous injection, the uptakes of the liver and spleen were about 35% and 25% ID/g, respectively. The circulation pathway of SWNTs was injected through the tail vein, circulated from the lung to the kidneys, and then accumulated in the organs of MPS. Additionally, luminance-enhanced semiconductor SWCNTs with a radius between 0.96-1.24 nm by laser vaporization (LV) and chemical enrichment could reach the NIR-IIb window with the wavelength of 1500-1700 nm under the excitation of 808 nm 115. The experimental results displayed that this system could realize the capillary imaging with the width and depth of 3 mm × 3 mm (Figure 5B) on the 7 days p.i. as well as a half-life of the blood circulation of 5.6 hours (Figure 5C). Except for the tumor uptake, SWNTs were mainly aggregated in the liver and spleen (Figure 5D), indicating that the SWNTs were mainly cleared through the hepatobiliary pathway for their unique shapes.

#### 3.1.2. Quantum dots

As a kind of semiconductor fluorescent nanoprobe, QDs have the advantages of good photochemical stability, narrow emission wavelength, and tunable fluorescence imaging characteristics. The ability to cross-linking molecules makes QDs potentially carrying drugs or other targeted molecules. In 1998, Bruchez* et al.* firstly applied QDs as fluorescent nanoprobes for biological staining and diagnosis, which set a milestone for the research of QDs in the biomedical field 148. QDs, as NIR-II contrast agents, comprise II~VI (CdTe/HgTe) 149-151, III~V (Inp/InAs/GaAs) 152-154 and IV~VI (PbS/PbSe) 155. Their applications in biological fields have been severely limited for a long time due to toxic ions, such as Cd^2+^, Hg^2+^, Pb^2+^, and As^3+^ 156. Studies showed that the preparation of QDs with the elements from the groups I to VI (Ag_2_S, Ag_2_Se, and Ag_2_Te) 116-118, unlike the toxic heavy metals such as Cd, Pb, and Hg, can endow them with good biocompatibility, excellent optical properties with NIR-II fluorescence, high quantum yield (QY) and light stability 157, 158.

**Figure 6:**
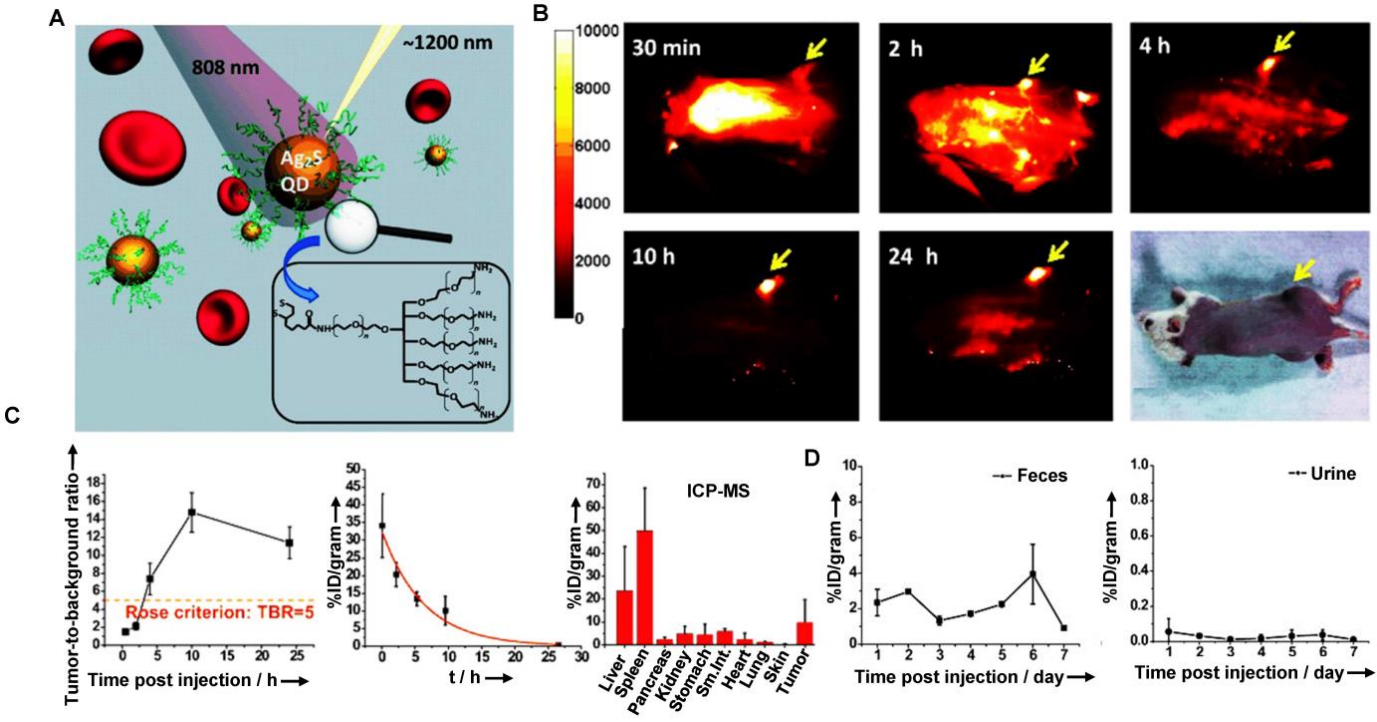
** Clearance of 6PEG-Ag_2_S QDs.** (**A**) Scheme of the 6PEG-Ag_2_S QDs with emission at 1200 nm upon 808 nm excitation. (**B**) NIR-II fluorescence imaging of 6PEG-Ag_2_S QDs in a xenograft 4T1 tumor-bearing mice. (**C**) The tumor-to-background ratio (TBR) plotted as a function of time p.i. for the NIR-II images, the representative plot of the %ID/gram of the 6PEG-Ag_2_S QDs in the blood after tail-vein injection, and the quantitative biodistribution of 6PEG-Ag_2_S QDs in various organs and tumor at 72hours p.i. (**D**) Short-term retention and clearance study of 6PEG-Ag_2_S QDs measured by ICP-MS. (reproduced from ref. 116 with permission from Wiley-VCH Verlag GmbH).

The clearance of QDs is mainly related to the injection dose, surface charge, and particle size. The clearance is negatively correlated with the dose. For instance, a large dose of QDs is mainly cleared from renal and hepatobiliary clearance pathways 159. Additionally, more charge-positive QDs were cleared through feces than charge-negative QDs. While the particle size increased from 5 nm to 25 nm, the clearance percentage of kidney and feces decreased significantly, and the fecal clearance rate of QDs with 5 nm was about twice that of 15-25 nm 159. Notably, macromolecules could enter the bile from the intrahepatic bile duct epithelial cells and hepatocytes *via* receptor-mediated endocytosis and then be cleared by the biliary tract. Besides, the uptake of QDs by MPS might concentrate QDs near the liver cells and enhance their biliary clearance. Based on the above considerations, take Ag_2_X (X=S, Se, Te) QDs as examples, Dai group has prepared the hydrophilic Ag_2_S QDs by a ligand exchange method, which could be used as NIR-II probes for cell imaging with good biological compatibility and imaging effect (Figure 6A) 116. The NIR-II fluorescence of 6PEG-Ag_2_S QDs was stably enhanced in the tumor region, and the accumulation in the tumor reached an unprecedented degree (> 10%ID/g) with an excellent imaging effect (Figure 6B). The half-life of the 6PEG-Ag_2_S QDs in blood circulation was 4.37 ± 0.75 hours, indicating that the modification of PEG efficiently avoids the opsonization of protein and the rapid uptake of MPS (Figure 6C). Moreover, the HD of 6PEG-Ag_2_S QDs was about 26.8 nm, larger than the renal filtration cut-off value (6 nm). Therefore, the 6PEG-Ag_2_S QDs were mainly accumulated in the liver and spleen at 72 hours p.i. in Balb/c mice, and then these QDs were primarily cleared through the biliary tract at a stable rate (Figure 6D).

**Figure 7:**
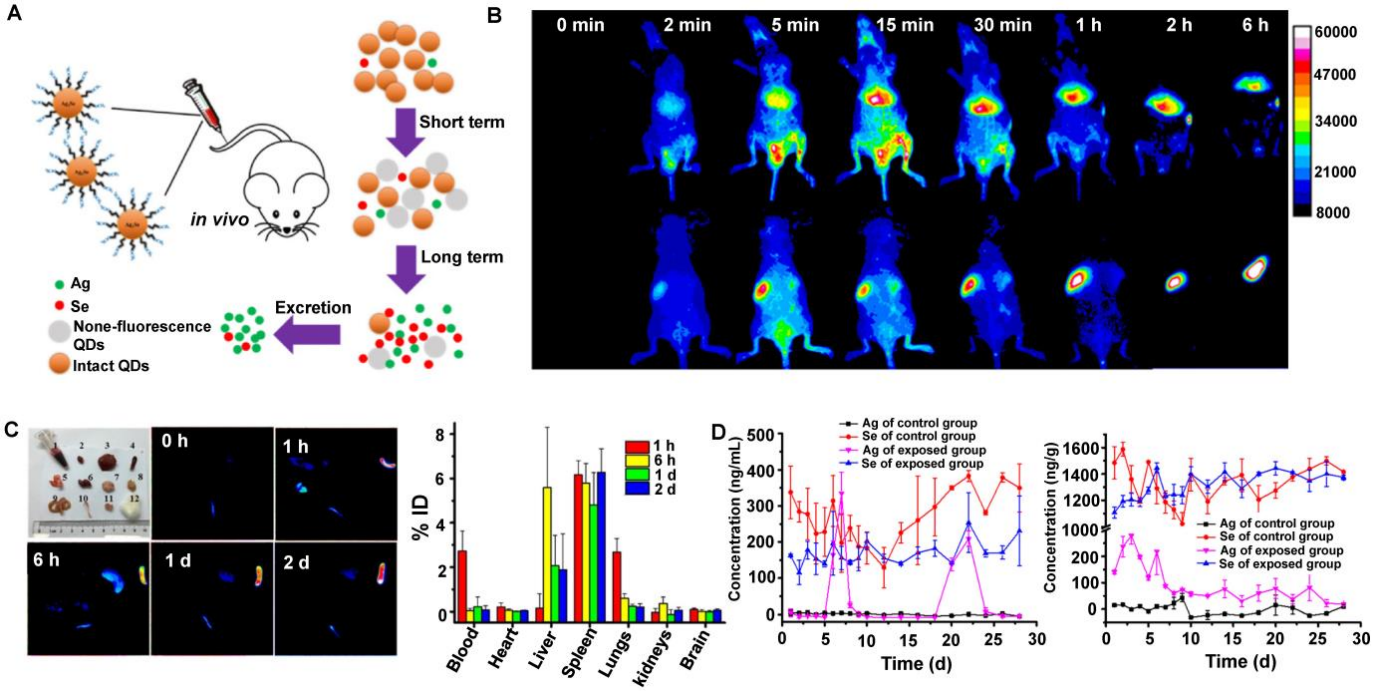
** Clearance of Ag_2_Se QDs.** (**A**) Scheme showing the preparation process of clearable ultrasmall Ag_2_Se QDs. (**B**)* In vivo* fluorescence imaging of Ag_2_Se QDs-PEG in nude mice within 6 hours after intravenous injection. (**C**) *Ex vivo* fluorescence images of Ag_2_Se QDs-PEG in tissues after intravenous injection and the distribution of Ag_2_Se QDs-PEG in mice based on tissue fluorescence intensity (% ID). (**D**) Clearance of Ag_2_Se QDs-PEG from mice (reproduced from ref. 117 with permission from American Chemical Society).

Similarly, Liu group has demonstrated that Ag_2_Se QDs with the advantages of deep penetration depth and negligible background had broad application prospects for *in vivo* imaging (Figure 7A, B) 117. The TEM observation and HD of Ag_2_Se QDs-PEG were 5.0 ± 0.8 nm and around 29.6 ± 5.1 nm, respectively. The coating of 5000 MW PEG and slight aggregation might be why the size increased. In addition, the charge of Ag_2_Se QDs-PEG is negative with -13.4 mV. Nevertheless, clearance behaviors in nude mice showed that Ag_2_Se QDs-PEG prefers to accumulate in the spleen and liver but was almost transformed and cleared within one day (Figure 7C). The total clearance rates of Ag in urine and feces were 12.7% ID and 14.5% ID on the 28th day, respectively. Interestingly, the Ag released from Ag_2_Se QDs-PEG was cleared by feces and urine, whereas the essential element Se was hardly cleared (Figure 7D). Moreover, a recent study has shown that the glucose-functionalized Ag_2_Se QDs (Glc-Ag_2_Se QDs) could be mainly cleared *via* renal clearances and eliminated from the body within 7 days for the small HD of 3.6 ± 0.3 nm 160. Furthermore, the same kind of Ag_2_Se QDs conjugated with the peptide octreotate (TATE) (Ag_2_Se-TATE) with an HD of 4.5 ± 2.5 nm could also be quickly renal cleared and maintain good tumor imaging capability 161. In a word, the various surface modifications endow QDs with unique sizes determined different clearance pathways, guiding rational design of clinical transformation.

Remarkably, a study has confirmed that the NIR-II fluorescent probe Ag_2_Te QDs showed an ideal tumor imaging effect after intravenous injection into mice 118. The zeta potential of Ag_2_Te QDs was measured to be around -4.6 mV. The negative charge effectively prevents the Ag_2_Te QDs from accumulation, enabling them to maintain long-term stability in water, making them more suitable for cellular uptake, and contributing to the clearance from the body. Besides, after intravenous post-injection, a great majority of Ag_2_Te QDs were accumulated predominantly in the MPS, and the fluorescence signal of Ag_2_Te QDs significantly reduced in the liver and spleen. Noticeably, Ag_2_Te QDs have an ultra-small hydrodynamic size of 4.3 nm measured by DLS, smaller than the renal filtration threshold, and could be cleared by the renal clearance pathway. Interestingly, Ag_2_Te QDs showed high crystallinity in urine, indicating that Ag_2_Te QDs may be cleared directly through urine without biodegradation, which is attributed to their ultrasmall particle size and low solubility product. According to these results, Ag_2_Te QDs could be efficiently excreted by the hepatic and renal elimination pathway. Therefore, the small size of the imaging probes may be the key factor in providing the efficient renal clearable ability of the nanoprobes.

#### 3.1.3. Rare earth materials

Rare earth materials consist of lanthanide ions embedded within an inorganic crystalline host matrix and contain multiple lanthanide ions, which work as sensitizer ions to absorb certain light wavelengths or as activator ions for emitting fluorescence 28, 162. Naczynski *et al*. firstly evaluated the potential applications of rare earth materials *in vivo* SWIR imaging 163. It is well known that the emission centers of rare earth contrast agents in the NIR-II region consist of five rare earth elements (Nd^3+^, Tm^3+^, Pr^3+^, Ho^3+^, Er^3+^) that can be excited by 808 nm or 980 nm laser. However, rare earth contrast agents still use Nd^3+^ or Er^3+^ as excitation centers 164. Moreover, rare earth contrast agents have the advantages of long storage time, long emission wavelength, highly controllable size, and lightless bleaching good biocompatibility, which has been widely researched 165, 166. However, most rare earth contrast agents cannot meet the ultra-small renal threshold. Therefore, the surface modifications of the rare earth contrast agents were very important to increase the clearance rate from MPS and reduce the potential toxicity *in vivo*. Studies also have shown that PEG could prolong the blood circulation time, contributing to nanoparticle escape and reducing the accumulation in MPS 80, 167, 168.

**Figure 8:**
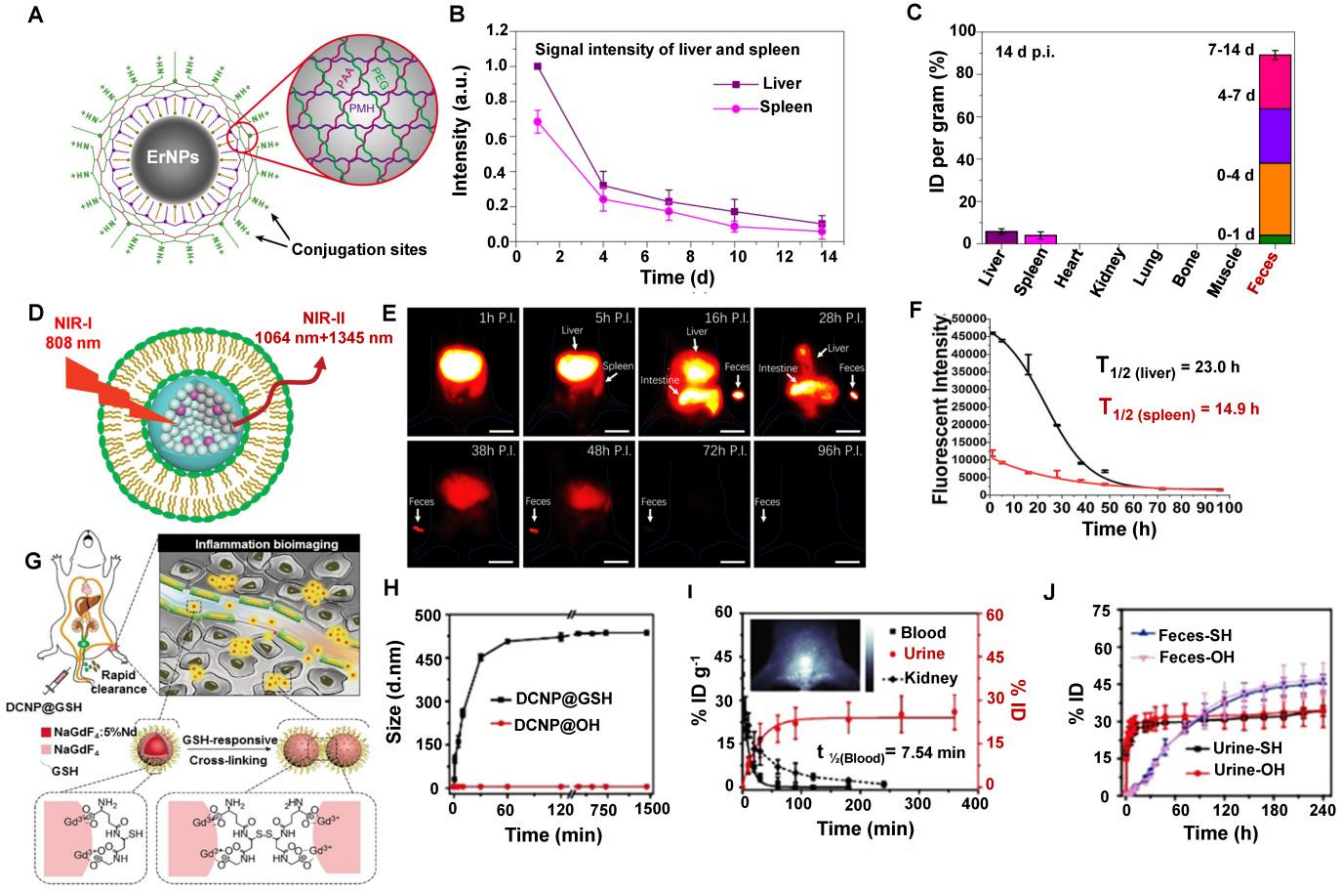
** Clearance of rare earth materials.** (**A**) Scheme of the hydrophilic ErNPs with crosslinking polymeric layers and amine groups on the surface as conjugation sites. (**B**) The clearance of ErNPs from the liver and spleen *via* plotting the signal intensity within two weeks. (**C**). Biodistribution of ErNPs in main organs and feces of ErNPs-treated mice at 14 days p.i. (reproduced from ref. 119 with permission from Springer Nature). (**D**) Schematic structure of RENPs@Lips. (**E**) Selected time points of *in vivo* NIR-II imaging from the whole-body and representative feces samples after intravenous injection of Rare earth contrast agents@Lips. (**F**) Quantitative analysis of NIR-II fluorescence intensity *in vivo* (liver and spleen region) (reproduced from ref. 120 with permission from Wiley-VCH Verlag GmbH). (**G**) Scheme of the NIR-II bioimaging for the acute local epidermal inflammation in the mice after intravenous injection of the ultra-small DCNP@GSH nanoprobes. (**H**) DLS results of DCNP@GSH and DCNP@OH in H_2_O_2_-containing mouse serum. (**I**) Pharmacokinetics of DCNP@GSH in the blood and urine clearance after intravenous administration. (**J**) Clearance of DCNP@GSH and DCNP@OH in urine and feces (reproduced from ref. 121 with permission from Wiley-VCH Verlag GmbH).

The nanoparticle form of rare earth is widely used in NIR-II *in vivo* imaging. For example, Dai group has developed the biocompatible cubic phase (α phase)-based rare earth nanoparticles (α-ErNPs: α phase NaYbF_4_:2%Er,2%Ce@NaYF_4_ nanocrystals) that showed bright downconversion fluorescence at about 1600 nm, which could be used for dynamic imaging of the tumor immunotherapy after bounding with anti-PD-L1 mAb in mice 119. The ErNPs were covered with the hydrophilic cross-linked coating (four layers: the inner layer is PMH, the second layer is 8Arm-PEG-NH_2_, the third layer is PAA, and the outermost layer is a mixture of mPEG-NH_2_ and 8Arm-PEG-NH_2_) with an average size of the ErNPs of ~35.5 nm in aqueous solution measured by DLS (Figure 8A). The biological distribution and clearance of ErNPs in the major organs and blood vessels in Balb/c mice showed that the fluorescence signals in the liver and spleen gradually increased within 24 hours p.i. (Figure 8B). Subsequently, a long period of strong ErNPs fluorescence signals was observed in feces (Figure 8C), indicating that the ErNPs were cleared by the biliary tract clearance pathway and confirming that about 90% of the injected α-ErNPs were cleared from the body within two weeks. This rapid and high level of ErNPs clearance can efficiently promote the clinical transformation of NIR-II contrast agents.

Liposomes have been considered Food and Drug Administration (FDA)-approved carriers in drug delivery with favorable biocompatibility and biodegradability 80. Another surface modification of the rare earth contrast agents (RENPs@Lips) was synthesized by the liposome-encapsulated NIR-II rare earth nanoparticles (HD:105 ± 5.2 nm) (Figure 8D) 120. In addition, the zeta potential of the nanoparticle was about -30 mV both in DMEM+FBS and in plasma, confirming the good physicochemical stability *in vivo.* RENPs@Lips exhibited an outstanding performance that can intraoperatively identify the orthotopic tumor vessels and embolization surgery by NIR-II imaging navigation, and the half-lives of RENPs@Lips in the liver and spleen were 23.0 hours and 14.9 hours (Figure 8F), respectively. Beyond all doubt, the intense signals in feces indicated that the RENPs@Lips were cleared from the liver to the intestines (bile to feces)* via* the hepatobiliary pathway (Figure 8E). More than 90% of RENPs@Lips could be eliminated from the body through the hepatobiliary system within 72 hours p.i. The advantages of superior biocompatibility, excellent cleanability, and competitive optical properties make RENPs@Lips a novel kind of promising NIR-II nanoparticle for clinical transformation.

To optimize signal-to-noise ratio (SNR) capability for long-term bioimaging and reduce the possible biotoxicity of accurate* in vivo* inflammatory imaging. Zhang group has reported a ROS-responsive cross-linking strategy for detecting inflamed areas with sub-10 nm *in vivo* 121. Lanthanide-based NIR-II fluorescent downconversion nanoparticles (DCNPs) were modified with glutathione (GSH) (Figure 8G), possessing the advantages of excellent photostability, high spatiotemporal resolution, and deep-tissue penetration. The ultrasmall size of 4.38 ± 0.57 nm less than renal cutoff-value endowed NaGdF_4_: 5%Nd@NaGdF_4_ DCNPs in favor of *in vivo* application. The nearly neutral surface (0.667 mV) ensures low MPS uptake and long-term circulation in the blood. Moreover, the stability of DCNP@GSH nanoprobes was identified with the constant HD of 6 nm during the incubation with mouse serum (37 °C, 24 h). Of note, after introducing the H_2_O_2_ into DCNP@GSH-containing mouse serum, the HD of DCNP@GSH increased to 451.5 nm within 30 minutes, indicating a successful cross-linking (Figure 8H). The cross-linking accelerated and enhanced the accumulation of these nanoprobes in the inflamed area in ICR mice because of the increased size and low clearance rate. Unsurprisingly, the inflamed tissue with a good SNR of around 3.5 at 10 minutes post-injection. The nearly neutral surface of DCNP@GSH ensured low MPS uptake and long-term circulation in the blood. Moreover, more than 22% ID of the injected DCNP@GSH was cleared from the body within 90 minutes p.i. by renal clearance on account of the 6 nm size with ROS-response (Figure 8I). And about 80% of DCNP@GSH was cleared out of the body through urine and feces within two weeks (Figure 8J). This novel strategy provides a new way for the efficient NIR-II bioimaging of *in vivo* inflammation with fast clearance.

**Figure 9:**
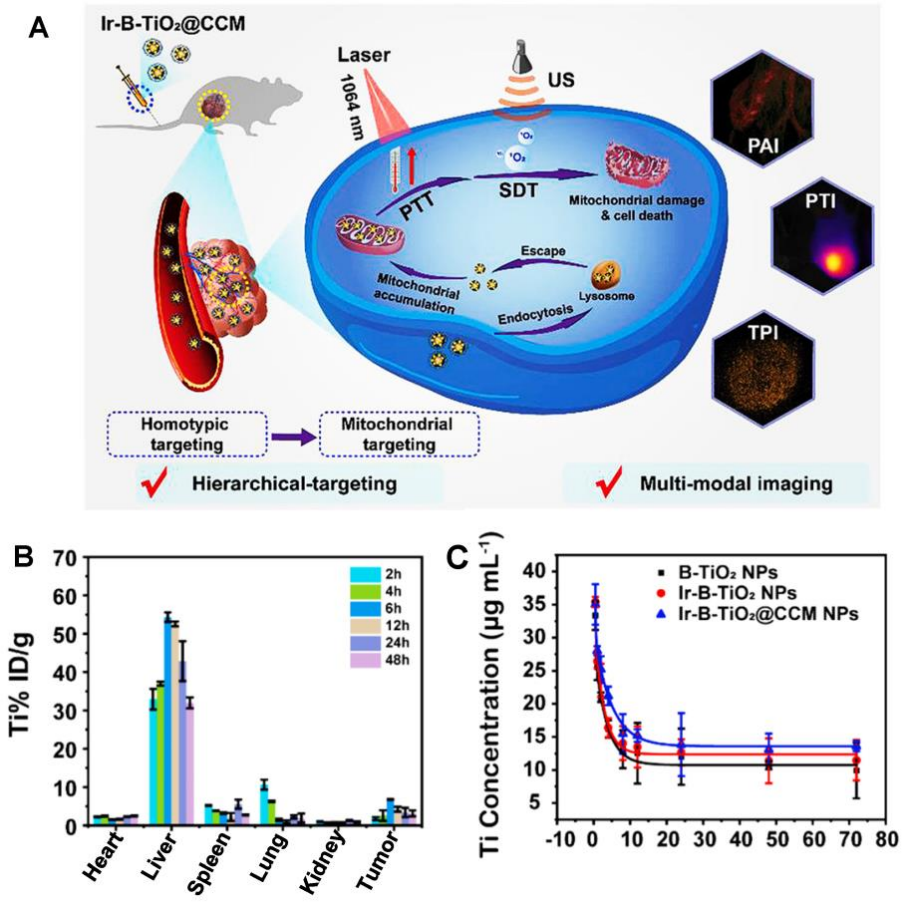
** Clearance of Ir-B-TiO_2_@CCM.** (**A**) The synthesis and mechanism of action of Ir-B-TiO_2_@CCM for multifunctional theranostic NIR-II photothermal/sonodynamic imaging and therapy. (**B**) Biodistribution after intravenous injection of Ir-B-TiO_2_@CCM for various time intervals (from 2 hours to 48 hours) is determined as the amount of titanium per gram of tissue by ICP-MS. (**C**) Blood-circulation time after intravenous injection of B-TiO_2_, Ir-B-TiO_2_, or Ir-B-TiO_2_@CCM (reproduced from ref. 123 with permission from Elsevier).

Recently, more and more evidence has shown that PEG phospholipid micelles could effectively avoid the uptake of MPS and promote the clearance of contrast agents through the hepatobiliary pathway 169-171. Unlike most contrast agents metabolized by organs such as the liver or kidney, RENPs@DSPE-mPEG could be cleared through circulating leukocyte internalization that belongs to the MPS pathway, which provides the possibility for improving the diagnostic and therapeutic effects of the tumor-targeting nanoparticles for clinical application. Cheng group has developed a DSPE-mPEG-encapsulated rare earth doped nanoparticle NIR-II contrast agent (RENPs@DSPE-mPEG) for imaging bone, blood vessels, and thrombosis with fast clearance, highlighting the potential for clinical transformation 122. The highly water-soluble monodispersed RENPs@DSPE-mPEG exhibited a dual peak fluorescent emission at ∼1064 nm (NIR-II) and 1345 nm (NIR-IIa), with fluorescence quantum yields of 8.2% and 4.6%, respectively. The zeta potential of the RENPs@ DSPE-mPEG was stable with -35 mV in DEME with FBS during 24 hours, and the hydrodynamic size was determined as 126.7 ± 1.5 nm by DLS analysis in PBS buffer. The clearance kinetics of RENPs@DSPE-mPEG in C57BL/6 mice showed that RENPs@DSPE-mPEG was concentrated in the liver at the beginning and cleared from the liver, bone, and spleen within 25 hours. The spleen was visible at 37 hours, and the tibia was the brightest at 165 hours. All the NIR-II probes were cleared from the body within two weeks. Although applying this probe for clinical transformation is still a great challenge, it can be used for *in vivo* bioimaging and contribute to developing and researching drugs and basic medical sciences. As an excellent method, cell membrane coating nanotechnology endows nanoparticles with unique properties and displays excellent translational potential in cancer diagnosis and therapy 123, 172. For example, another nano-platform of rare earth was put forward that the functionalization of black-titanium nanoparticles with iridium complexes and cancer cell membranes (Ir-B-TiO_2_@CCM) for synergistic sonodynamic cancer imaging and photothermal therapy (Figure 9A) 123. Upon functionalization with Ir and camouflaging with CCM, the zeta potential of the particles drastically changed to 14.82 ± 1.98 mV and finally to -4.38 ± 2.99 mV. As an imaging contrast agent, the nanoparticles with 389.15 ± 4.04 nm measured by DLS accumulated at the tumor site with a high spatial resolution by the enhanced permeability and retention effect (EPR) upon synergistic irradiation at 1064 nm and ultrasound irradiation. As a therapeutic agent, the particles were proven to produce heat under 1064 nm laser irradiation efficiently and catalytically form ROS with ultrasound irradiation. According to the biodistribution of Ir-B-TiO_2_@CCM *in vivo*, a majority of the particles accumulated in the liver by ICP-MS, and a significant amount of signals also accumulated in the tumor (Figure 9B). Meanwhile, the half-time for Ir-B-TiO_2_@CCM was about 3.31 hours (Figure 9C). Although with a negative charge, the Ir-B-TiO_2_@CCM was mainly cleared by the hepatobiliary pathway owing to its large size.

#### 3.1.4. Other inorganic contrast agents

Besides the materials mentioned above, other inorganic materials with NIR-II optical properties have also been studied for *in vivo* imaging with the development of materials and achieved great achievements, such as liquid metal-based nanoplatforms (liquid metal@SiO_2_-RGD), Black Phosphorus nanospheres (BP@Lipid-PEG), gold (Au) cluster, Carbon dots(CDs) 29, 84, 119, 173, 174.

**Figure 10:**
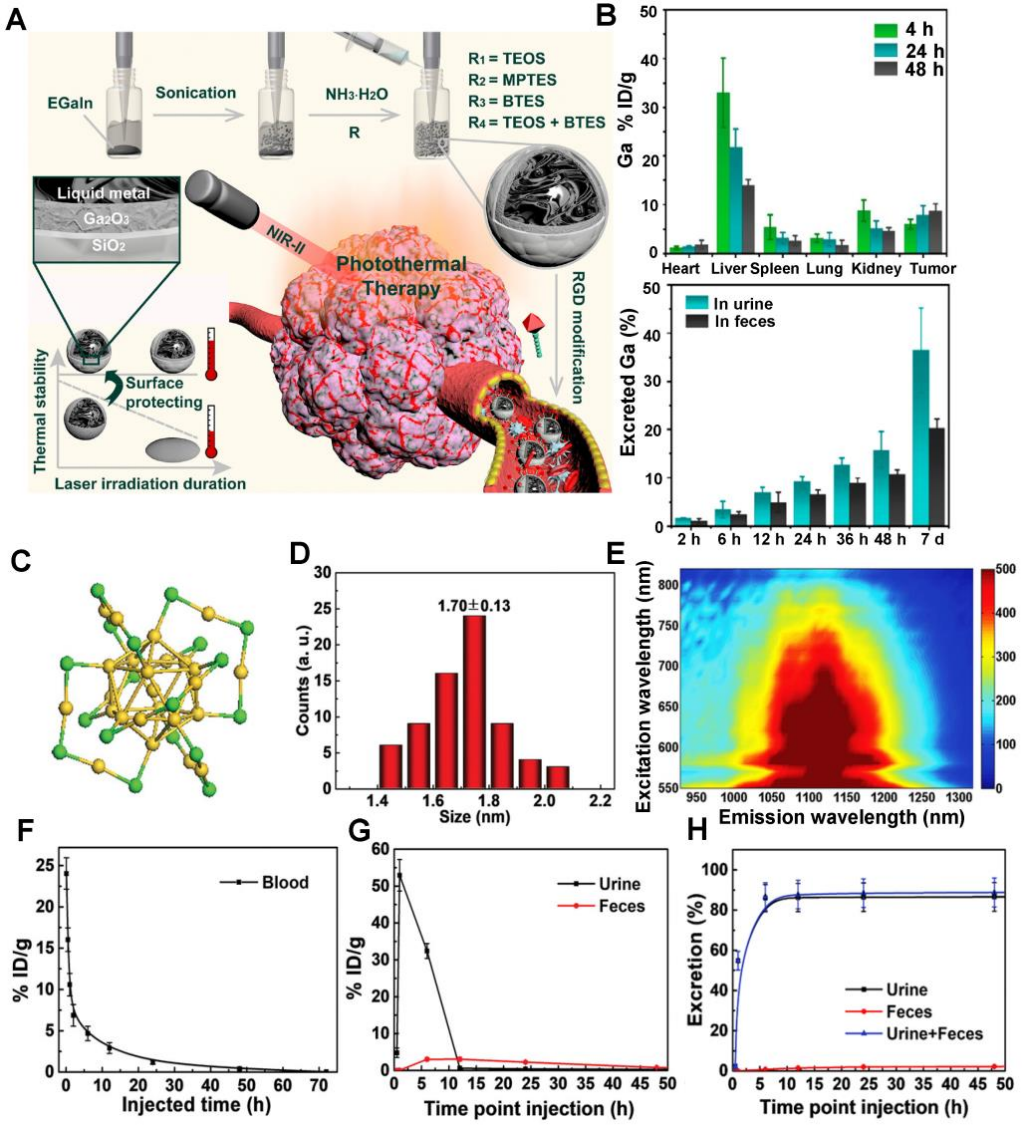
** Clearance of other NIR-II inorganic contrast agents.** (**A**) The stepwise synthesis of liquid metal@SiO_2_-RGD targeted core/shell nanoparticles, detailed composition/nanostructure, unique functionality for tumor-targeted accumulation, and subsequent photothermal tumor hyperthermia in the NIR-II window. (**B**) *In vivo* biodistribution of Ga in major organs and tumors as well as accumulated Ga in feces and urine cleared out of the mice body (reproduced from ref. 124 with permission from American Chemical Society). (**C**) The crystal structure of gold clusters has 25 gold atoms as the core and 18 sulfur atoms. (**D**) Size of gold clusters detected with TEM. (**E**) Photoluminescence (PL) versus excitation spectra of gold clusters with an emission center of 1120 nm. (**F**) Blood half-time of gold clusters determined by ICP-MS. (**G**) ICP-MS measurement of the time-dependent concentration of gold clusters in urine and feces. (**H**) Cumulated urine, feces, and total (urine and feces) clearance of gold clusters with collected time within 48 hours (reproduced from ref. 125 with permission from Wiley-VCH Verlag GmbH).

Recently, Shi group confirmed that the surface chemistry modifications of liquid metal-based nanoplatforms with an inorganic silica nanoshell provided NIR-triggered photothermal hyperthermia for cancer therapy with highly tumor-targeting in the NIR-II region (Figure 10A) 124. After continuous surface modification, the zeta potential and the average HD of liquid metal@SiO_2_-RGD were about 20 mV and 236.43 nm, respectively. The half-life of liquid metal@SiO_2_-RGD in U87 tumor-bearing mice was 1.04 hours. Notably, the cumulative clearance of Ga increased to 55.7% in total after intravenous injection of liquid metal@SiO_2_-RGD for 7 days p.i. (Figure 10B), suggesting the gradual degradation of liquid metal@SiO_2_ during blood circulation and metabolism* in vivo*. The remarkable advantage of liquid metal@SiO_2_-RGD is that the inorganic SiO_2_ layer is too thin (about 3 nm in thickness) to resist the tumor microenvironment for biodegradation, which endows it with good biocompatibility for clinical transformation. Besides, Zhang group also presented a bright Au25 cluster with a unique cage-like structure emitting NIR-II fluorescence in the 1100-1350 nm region by the charge transfer between ligand and gold core (Figure 10C, E) 125. As a good NIR-II fluorescence, the Au_25_ cluster was applied for time-resolved brain blood-flow imaging, significantly differentiating the brain diseases between the injured and healthy mice *in vivo***.** Gold clusters with NIR-II fluorescence could be used to monitor high-resolution imaging at a depth of 0.61 cm in kidney. (Figure 10D). Of note, biosafety, especially the clearance pathway, is a key factor for *in vivo* imaging as a bioimaging agent. The half-time of gold clusters in blood was 52.3 minutes by determining the time-course gold concentration (Figure 10F). As shown in Figure 10G, the gold concentrations in urine collected after 1 hour and 2 hours were 32.2 and 53.0% ID g^-1^, respectively, and the highest gold concentration in feces was 3.1% ID g^-1^ after 6 hours with slow clearance. The cumulative clearance curves showed that 86.6% and 2.2% of gold clusters were cleared by urine and feces after 48 hours of intravenous injection, respectively (Figure 10H). With the ultrasmall size of 3.2 nm, the gold clusters can be rapidly cleared by renal clearance.

Additionally, carbon dots (CDs) with high photostability, low biotoxicity, and well-controlled size are remarkable imaging agents for* in vivo* bioimaging and have been obtained widely concerned. Recently, Hao group has reported that watermelon-derived CDs with 900-1200 nm emission with high QY (0.4%) and good biocompatibility and has been proven to be efficient probes for NIR-II bioimaging *in vivo* 126. Of note, owing to the small size of 6 nm, moreover, the surface charge of CDs was also investigated by zeta potential analysis, indicating the negative charge nature on the surface (-4.9 mV), demonstrating that approximate 65% of CDs were cleared from the urine during 6 hours. These findings opened up a new way to develop a multifunctional CD-based phototheranostics platform for integrating the *in vivo* NIR-II bioimaging and photothermal therapy (PTT) of cancer therapy. Another reported NIR-II inorganic contrast agent is Black phosphorus (BP) with a narrow band gap semiconductor. Li group has reported that the BP nanoparticles modified with cholesterol could be encapsulated with the PEGylated lipid to form BP@lipid-PEG nanospheres for NIR-II imaging *in vitro* and *in vivo* 84. Moreover, BP@lipid-PEG nanospheres with large size of about 97.7 ± 27.8 nm were rapidly cleared from blood circulations and easily accumulated in the liver. The signal intensity of BP@lipid-PEG in the liver was acquired at 24 hours p.i. and was largely decreased to 47.6%. Interestingly, accompanied by the decrease of the fluorescence signal in the liver, the fluorescence signal of the spleen was increased and achieved the highest at 24 hours p.i., illustrating the clearance of BP@lipid-PEG nanospheres mainly through the hepatobiliary clearance pathways.

In a word, NIR-II inorganic contrast agents, including single carbon nanotubes, QDs, rare earth, etc., are often insoluble in water for physiological applications. Therefore, most of the reported NIR-II inorganic contrast agents must be modified with water-soluble groups or physical wrapping for the application of *in vivo* imaging. Besides, most inorganic substances cannot be discharged from the physiological environment *in vivo* because of their limited particle size and lack of degradability. In general, if the average size of the inorganic nanoparticle materials (INPMs) is small enough (< 6 nm), they can be cleared into the urine through the kidneys to a large extent. However, synthesizing ultra-small INPMs is difficult. Moreover, INPMs require a relatively large particle size to ensure appropriate fluorescence intensity. Except for ultra-small size INPMs, most INPMs are cleared through hepatobiliary clearance, which takes a long time and causes long-term toxicity concerns for INPMs 175, 176. Therefore, optimizing inorganic clearance focuses on two critical points: i) exploring degradable or renal scavenge INPMs; ii) optimizing clearance by specific surface modification 177.

To minimize the biotoxicity of inorganic materials, modifications are necessary for better applications *in vivo*, such as Lips, DSPE-mPEG, GSH, and cancer cell membranes. And the clearance of NIR-II inorganic contrast agents is related to many factors, such as size, charge, surface modification, and composition. SWCNT, as NIR-II dye, has a good imaging effect *in vivo*. The unmodified SWCNTs showed obvious uptake in MPS, attributing to the lipophilicity of carbon atoms, and mainly cleared through the hepatobiliary pathway. Size is a critical factor for clearance. For example, the large-sized 6PEG-Ag_2_S is mainly cleared through the biliary tract, while the small-sized Ag_2_Te can be cleared through the hepatobiliary and kidneys. It is worth nothing that Ag in Ag_2_Se-PEG can be cleared from the biliary tract and urinary system while most of the essential element Se retains in the body. Although some agents with big size seems hardly cleared from the body, biodegradable modification is a good method to apply for current organic materials. For example, liquid metal@SiO_2_-RGD can gradually degrade and metabolize. Besides, natural materials, such as CDs, can improve biocompatibility for applications, and they can be rapidly cleared by urine due to their small size.

### 3.2. Clearance pathways of organic NIR-II contrast agents

#### 3.2.1. NIR-II contrast agents based on conjugated polymers

Conjugated polymers, a new type of optical material, have become a research hotspot because of their excellent optical properties and biocompatibility 178-183. However, most of the conjugated polymers are hydrophobic and with sizes ranging from 50 nm to several microns. Hence, most conjugated polymers cannot be cleared through the kidney system, leading to accumulation in the spleen or liver 184. If MPS cannot degrade these hydrophobic polymers, they will accumulate in organs for months to years and may cause long-time unforeseen biotoxicity 185.

**Figure 11:**
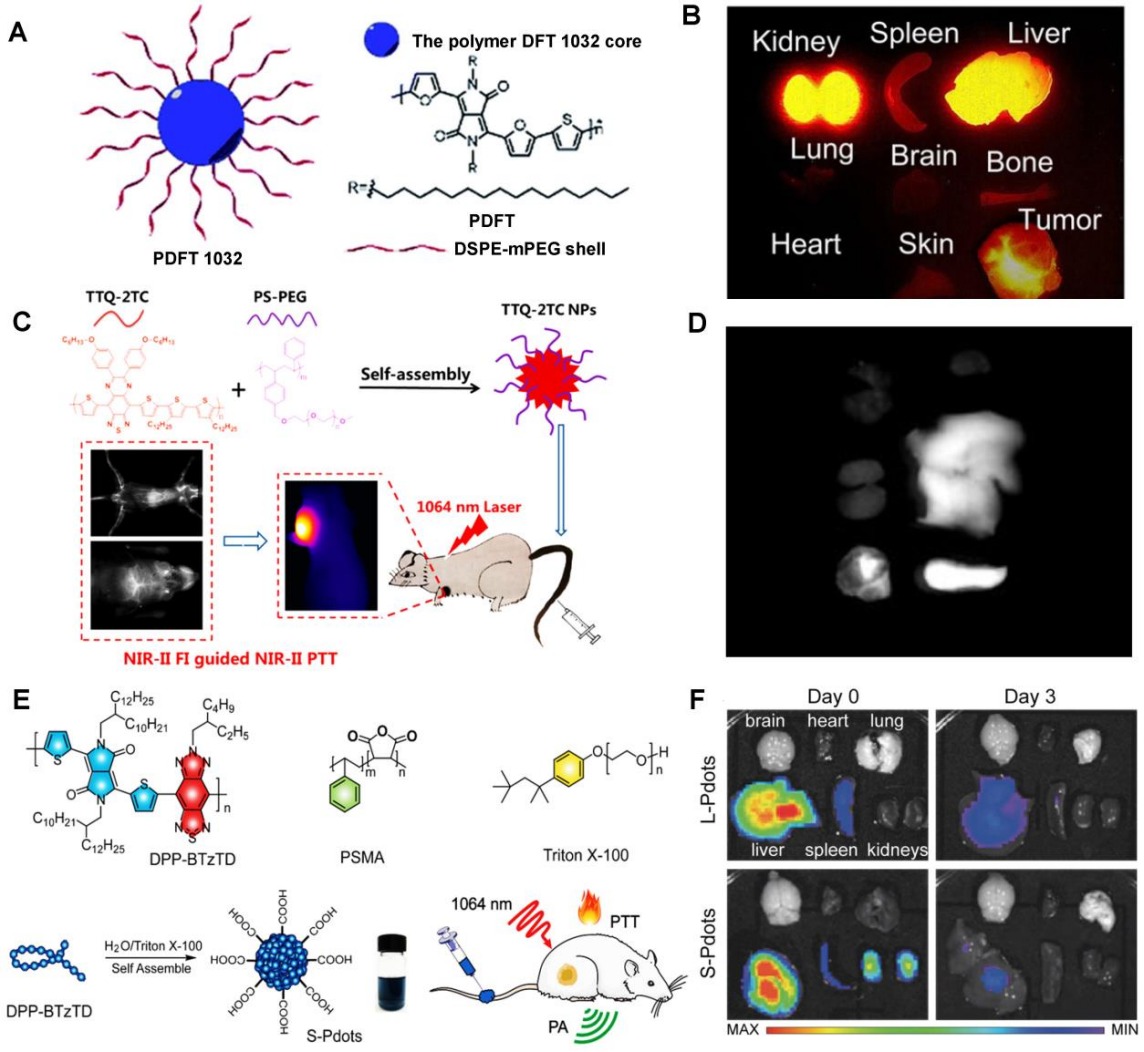
** Clearance of NIR-II contrast agents based on conjugated polymers.** (**A**) Schematic illumination for PDFT1032 nanoparticle consisted of semiconducting polymer DFT and a hydrophilic DSPEm-PEG shell. (**B**) The *ex vivo* biodistribution for different organs (reproduced from ref. 127 with permission from Royal Society of Chemistry)**.** (**C**) Chemical structures of the conjugated polymer, the purple region represents the quinoid polymer backbones, and the blue region represents the electron-withdrawing group. (**D**) *Ex vivo* NIR-II imaging of the vital organs (heart, liver, spleen, lung and kidney) (reproduced from ref. 128 with permission from American Chemical Society). (**E**) Chemical structural formula of the semiconducting polymer DPP-BTzTD, functional polymer PSMA, and Triton X-100. (**F**) NIR-II fluorescence imaging of various organs dissected from mice after the intravenous injection of S-Pdots at different time points p.i. (reproduced from ref. 129 with permission from Wiley-VCH Verlag GmbH).

For instance, Cheng group has developed a semiconducting polymer (named PDFT1032) based on furan-containing diketopyrrolopyrrole polymers (PDFT) with NIR-II emission peak at 1032 nm (Figure 11A) 127. Moreover, PDFT1032 has been applied in several important bioimaging scenarios, including tumor imaging for evaluating vascular embolotherapy on a subcutaneous osteosarcoma model, image-guided orthotopic tumor surgery, and sentinel lymph node biopsy. PDFT1032 is a potential NIR-II probe (HD: 68 nm) with high photostability and low toxicity *in vivo*. After 24 hours of intravenous injection of PDFT1032 into the mice, the dynamic imaging and biological distribution studies revealed that the nanoprobes were rapidly cleared by renal and hepatobiliary systems, contributing to obtaining a good NIR-II tumor imaging capability (Figure 11B), making it possible for NIR-II image-guided surgery. Similarly, Fan group has designed a conjugated polymer TTQ-2TC with optimized absorption in the NIR-II window (Figure 11C) 128. With the alkyl-side-chain-modified bithiophene (2TC) as the donor unit, excellent solubility in organic solvents and obvious NIR-II signals were obtained. Based on the conjugated polymer TTQ-2TC encapsulation in amphiphilic copolymer PS-PEG, the water-soluble TTQ-2TC NPs were formed for NIR-II imaging-guided phototherapy. Additionally, the NIR-II imaging of TTQ-2TC NPs was mainly observed in the tumor, liver, and spleen tissues rather than other organs at 24 hours p.i. (Figure 11D), indicating that TTQ-2TC NPs could be accumulated in the tumor mainly due to the strongly EPR effect, and for its large size of 140 nm, the primary metabolic pathway of NPs is the hepatobiliary system. Significantly, Yuan group has reported an absorbable and metabolizable NIR-II conjugated polymer (DPP-BTzTD PDots) 129. By a typical nanoprecipitation, conjugated polymer DPP-BTzTD was mixed with function polymer poly (styrene-*co*-maleic anhydride) (PSMA) in tetrahydrofuran (THF) and quickly injected into Triton X-100 solution under sonication in the ice-water solution for 10 minutes, and finally, DPP-BTzTD PDots were formed (Figure 11E). DPP-BTzTD PDots were used for photoacoustic (PA) image-guided PTT with advantages of ultra-small particle size, bright photoacoustic signal, good biocompatibility, excellent photostability, high photothermal conversion efficiency, and ablation capability. According to the size, Pdots can be divided into S-Pdots (average sizes of diameters, 4 nm) and L-Pdots (average sizes of diameters, 25 nm). The Zeta potential results revealed that both the S-Pdots and L-Pdots possessed a negative potential of -26 mV and -39 mV, respectively. The clearance data showed that L-Pdots mainly accumulated in the liver, while S-Pdots mainly accumulated in both liver and kidneys after 6 hours of intravenous injection. Interestingly, S-Pdots showed a faster clearance than L-Pdots, because L-Pdots were almost cleared from the body through the hepatobiliary clearance pathway, and S-Pdots can be cleared by two clearance systems of hepatobiliary and kidneys (Figure 11F). Although most conjugated polymers can easily lead to accumulation in the liver and spleen, however, ultra-small NIR-II conjugated polymers, such as S-Pdots, can also be cleared rapidly through the kidneys because of their sizes less than the renal clearance threshold, which greatly reduces the potential toxicity *in vivo*, thus providing a new idea for the development of NIR-II conjugated polymer fluorescent probes. Biodegradable NIR-II PA contrast agents have been widely concerned for good biocompatibility in cancer therapy 32, 186, 187. Interestingly, Pu group has firstly reported a series of biodegradable NIR-II PA contrast agents based on semiconducting polymer nanoparticles (SPN) the effective clearance *in vivo*. 32 Based on the FDA-approved hydrolyzable polymer matrx (PLGA-PEG) and the oxidizable thiophene moiety in conjugated backbones, the NIR-II SPNs, such as SPN-PT, SPN-DT, and SPN-OT, could be efficiently degraded with rich myeloperoxidase (MPO) and lipase, making the size of these nanoparticles decrease from 30 nm into ~1 nm. Because of the ultrasmall hydrodynamic sizes of degradation products, these NIR-II SPNs could be effectively cleared from living mice *via* both renal and hepatobiliary clearances within 15 days p.i.

In short, most NIR-II conjugated polymers with large sizes are quickly accumulated in MPS and tend to be cleared by MPS or hepatobiliary clearances. However, by rational design and modified nanoprecipitation, the metabolizable and highly absorbable NIR-II conjugated polymer S-pdots make up for this shortcoming. Another kind of biodegradable NIR-II agent is NIR-II SPNs that can be degraded in the presence of biologically abundant MPO and lipase, leading to the 30 nm nanoparticles dismembered to ultrasmall nanoparticles (~ 1 nm), greatly improved biological safety. Thus, the ingenious design and proper modification are very important for the clearance of NIR-II conjugated polymers.

#### 3.2.2. NIR-II contrast agents based on organic molecules

In order to obtain the absorption and emission in the NIR-II region, the contrast agents need a large conjugated molecular skeleton system to achieve a low Band-Gap. Usually, the light-emitting core is a hydrophobic conjugated molecular skeleton. Some strategies were proposed to obtain water solubility with water-soluble chemical groups: i) wrapped by amphiphilic polymer or protein cavity to form nanoparticle or fusion body; ii) modified by water-soluble polymer (PEG, Peptide, Aptamer, etc.); iii) modified by water-soluble ionic (sulfonic, phosphoric, ammonium, etc.), such as traditional Polymethine/Cyanine/Rhodamine through structural optimization to achieve the absorption or emission in the NIR-II region.

##### 3.2.2.1. NIR-II contrast agents encapsulated in amphiphilic matrixes

**Figure 12:**
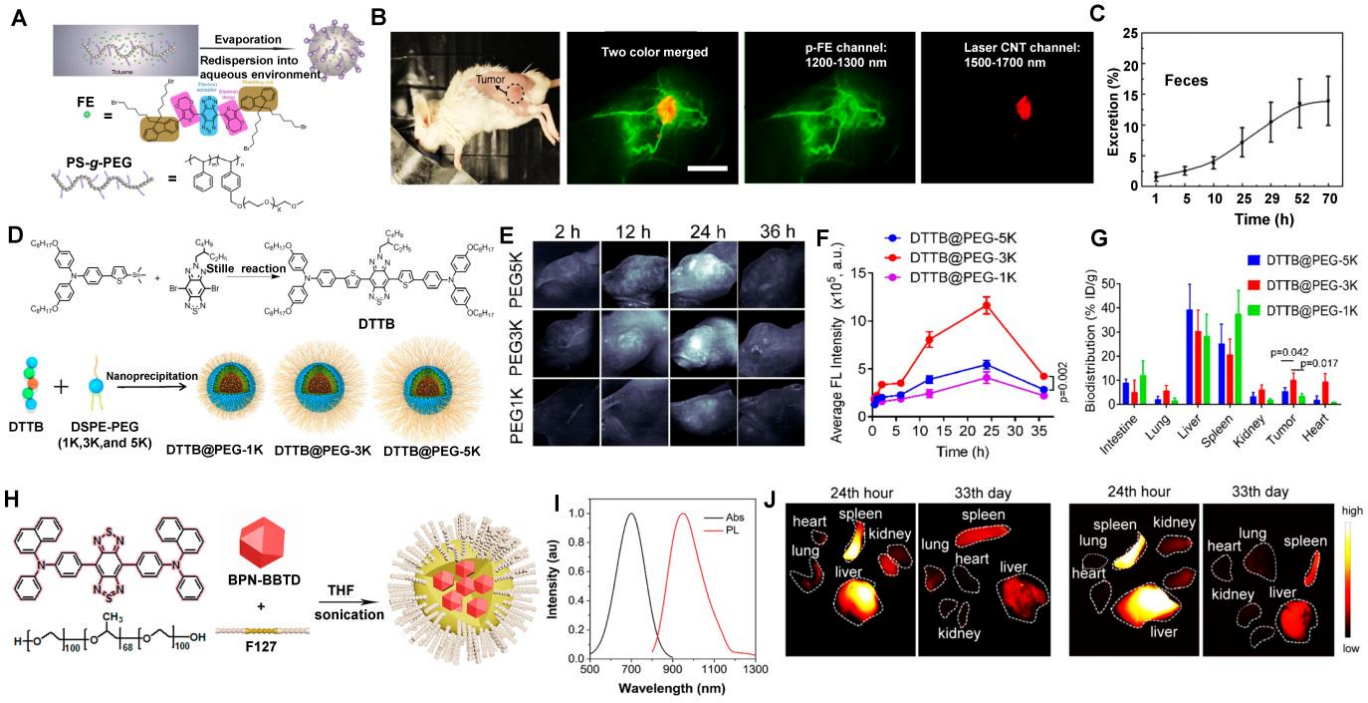
** Clearance of NIR-II molecular contrast agents** encapsulated in amphiphilic matrixes. (**A**) Schematic illumination of the p-FE synthesis, chemical structures of FE, and the PS-g-PEG polymer. (**B**) High-magnification of NIR-II fluorescence imaging of a 4T1 tumor in a mouse with two colors. (**C**) The clearance behavior of p-FE (reproduced from ref. 35 with permission from Springer Nature). (**D**) Schematic illustration of DTTB@PEG NMs preparation. (**E**) NIR-II fluorescence images of the 4T1 tumor-bearing mice obtained at different times p.i. (**F**) Semiquantitative analysis of the NIR-II fluorescence signals in 4T1 tumor regions at different times. (**G**) Biodistributions of DTTB@PEG NMs at 36 hours p.i. (reproduced from ref. 130 with permission from American Chemical Society). (**H**) Scheme of the synthesis and chemical structures of BPN-BBTD NPs. (**I**) Absorption and PL spectra of BPN-BBTD in chloroform. (**J**) Biodistribution of BPN-BBTD NPs in various organs of subcutaneous (up) and orthotopic tumor-bearing mice (down) (reproduced from ref. 131 with permission from American Chemical Society).

Amphiphilic matrixes are extensively used in NIR-II molecular contrast agents solubilization to get water-soluble properties 35, 131, 133, 186, 188-194. Dai group has developed a bright organic contrast agent (named p-FE) for bioimaging in the NIR-II region (Figure 12A) 35. As shown in Figure 12B, two-color fluorescence imaging in the NIR-II region in a mouse tumor model was performed. One color was p-FE emitting in 1100-1300 nm to highlight vasculatures, and another color was CNTs (carbon nanotubes) emitting in 1500-1700 nm to highlight the tumor. By overlaying these two colors, the fluorescence imaging showed an amount of fluorescence signals in vessels of the tumor. Due to the HD of around 12 nm, p-FE was primarily accumulated in the liver at 24 hours p.i., followed by the accumulation in the lung. Meanwhile, other organs (heart, spleen, kidney, bladder, and testis) were with low uptake. Ultimately, p-FE was gradually cleared through feces within 3 days, implying the hepatobiliary clearance pathway is the dominant clearance pathway owing to the large size over the renal cut-off value (Figure 12C).

By providing essential insights into the biodistributions of drugs *in vivo*, NIR-II fluorescence imaging plays a critical role in developing the preclinical drug with high spatiotemporal resolution and deep tissue penetration. Liang group developed NIR-II contrast agents modified with different PEG ligands at various chain lengths to monitor the metabolism of nanomedicines *in vivo* (Figure 12D) 130. The alternative D-A-D conjugated oligomer DTTB could achieve NIR-II emission peaked at 1050 nm with high QYs of 13.4%. DTTB-anchored nano-micelles (DTTB@PEG NMs) with different chain lengths have observed similar average HDs of around 80 nm determined by DLS with spherical morphologies. *In vivo* bioimaging results revealed that DTTB with an appropriate PEG chain length could be in favor of long blood circulation and excellent tumor targeting (Figure 12E, F). Significantly, in comparison with DTTB@PEG-1K and DTTB@PEG-5K, the DTTB@PEG-3K displayed the most signal enhancement at the tumor sites. *Ex vivo* NIR-II fluorescence imaging of DTTB@PEG NMs in the major organs and tumors showed the strongest fluorescence in the liver and spleen at 36 hours p.i., suggesting that these NIR-II contrast agents are mainly cleared from the hepatobiliary clearance pathway (Figure 12G).

NIR-II aggregation-induced emission luminogen (AIEgen) photosensitizers have been proven to have outstanding phototherapeutic effects and accuracy of image-guided surgery/PTT 133, 186-190, 195. Tang group has designed a molecular NIR-II emissive theranostic system (BPN-BBTD NPs) (HD: 37.1 ± 2.3 nm) by encapsulating BPN-BBTD with amphiphilic polymer F127 (Figure 12H, I) 131. For the biodistribution of BPN-BBTD NPs, strong signals of NIR-II fluorescence were predominantly accumulated in the liver and spleen after 24 hours p.i., and NIR-II fluorescence intensities decreased distinctly in the liver and spleen on the 33rd day, which implicated that BPN-BBTD NPs were degraded and eliminated from the body through the hepatobiliary pathway (Figure 12J). Similarly, Hong group has reported that nanoparticles (HLZ-BTED dots) comprised of NIR-II AIE fluorescence (HLZ-BTED) and DSPE-PEG were applied for *in vivo* bioimaging 132. Although the zeta potential of the HLZ-BTED dots was 16.4 mV, the HD of about 60 nm determined by DLS and an average size of 50 nm determined by TEM made it easily accumulate in the liver and spleen at 192 hours p.i., indicating that the clearance route of the HLZ-BTED dots was predominately through the hepatobiliary system. As an excellent NIR-II fluorescence probe, the fluorescence intensity in tumors reached the highest level at 48 hours after intravenous injection under NIR irradiation (808 nm, 90 mW/cm^2^). The NIR-II fluorescence signal of the AIE HLZ-BTED dots was selectively strong in tumors until 8 days p.i., significantly contributing to long-term tumor imaging and imaging-guided tumor therapy.

##### 3.2.2.2. NIR-II contrast agents encapsulated in or modified with proteins

**Figure 13:**
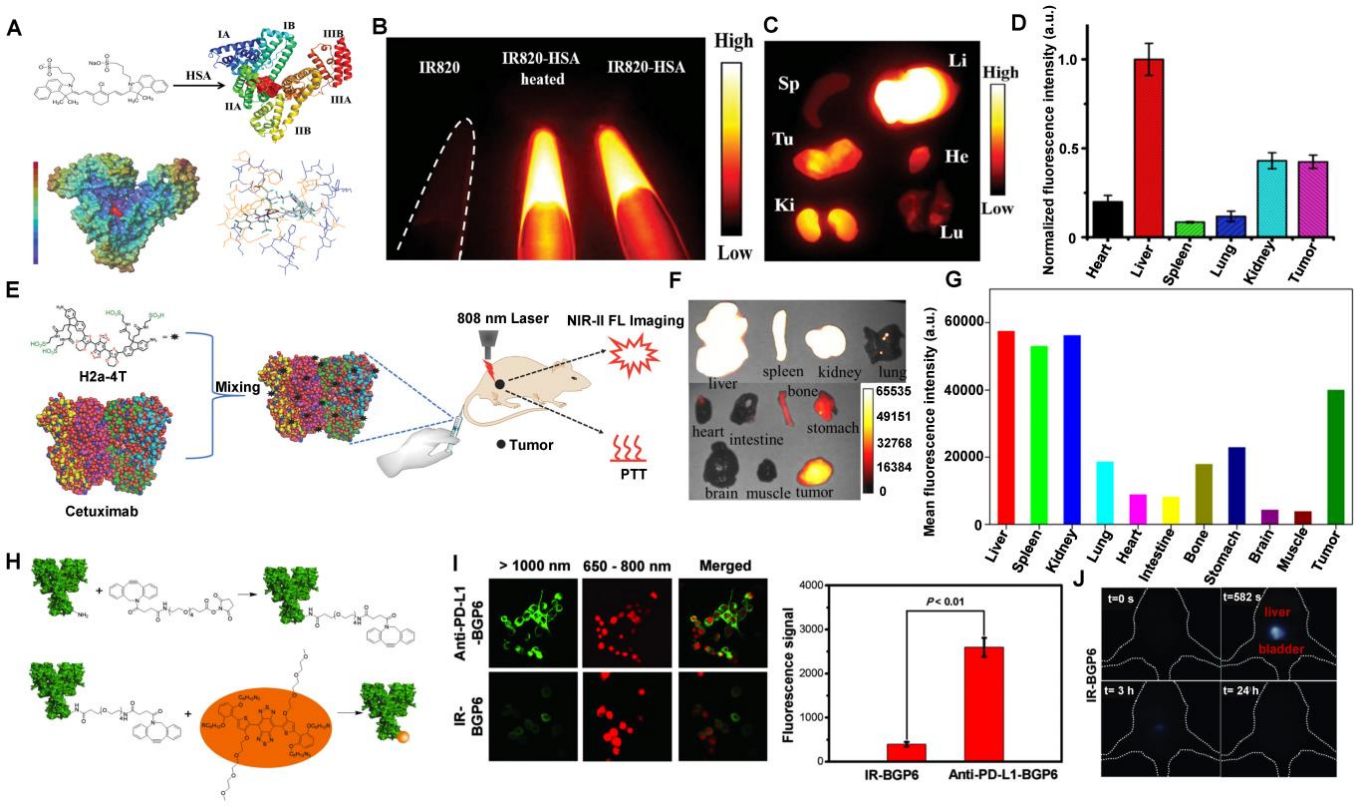
** Clearance of NIR-II contrast agents encapsulated in or modified with proteins.** (**A**) Schematic drawing of the interaction of IR820 with HSA. (**B**) NIR-II fluorescent image of IR820, IR820-HSA, and IR820-HSA heated at 60 °C. (**C**) The distribution of IR820-HSA in the major organs and tumors dissected from mice at 8 hours. (**D**) Semiquantitative analysis of fluorescence intensity of IR820-HSA imaging in different organs of 143B tumor-bearing mice (reproduced from ref. 133 with permission from Wiley-VCH Verlag GmbH). (**E**) Synthesis of H2a-4T@Cetuximab complex and the NIR-II fluorescence imaging and phototherapy. (**F**) *Ex vivo* fluorescence images of H2a-4T@Cetuximab in the main organs and tumors of HCT116 tumor-bearing mice at 24 hours. (**G**) Quantitative analysis of mean fluorescence intensity in different images of organs and tumors (reproduced from ref. 134 with permission from Wiley-VCH Verlag GmbH). (**H**) Schematic illumination of the combination of PD-L1 mAb and IR-BGP6. (**I**) Comparison of anti-PD-L1-BGP6 and IR-BGP6 for targeting the PD-L1 positive cancer cell line MC38 *in vitro*. (**J**) Clearance behaviors of the NIR-II fluorescence probe IR-BGP6 (reproduced from ref. 135 with permission from Wiley-VCH Verlag GmbH).

The development of novel metabolic and biocompatible contrast agents with a phototheranostics platform for efficient cancer therapy has attracted widespread attention in recent decades. NIR-II molecular contrast agents encapsulated in or modified with proteins could increase biocompatibility *in vivo* 34, 133, 134, 140, 190, 196, 197. Cheng group has reported that human serum albumin (HSA) proteins complexed with IR820, a sulfonated NIR-I organic dye, showed a significant 21-fold increase in fluorescence for NIR-II imaging 133. IR820 binding to HSA (MW: 66 kDa), could efficiently be applied for *in vivo* NIR-II imaging (Figure 13A, B). The feasibility of NIR-II fluorescence imaging with IR820-HSA in tumors was investigated in mice at 8 hours p.i. Except for the high NIR-II fluorescence intensity in the tumor, evident accumulation also showed in the liver, indicating that the probe was eliminated through the digestive system because its weight was far more than the renal filtration threshold of 40 kDa (Figure 13C, D).

Compared with the serum albumins, targeted proteins/peptide ligands endow NIR-II contrast agents for better molecular imaging and imaging-guided microsurgery 56, 66, 67, 198. For example, Hong group has reported a small-molecule NIR-II contrast agent H2a-4T complexed with cetuximab proteins possessing advantages of good water solubility, outstanding biocompatibility, excellent optical properties, and targeting ability (Figure 13E) 134. With binding to Cetuximab (Erbitux), a 152 kDa recombinant monoclonal antibody combined with the extracellular domain of EGFR, H2a-4T@Cetuximab complex could exhibit a remarkable cell-killing ability and achieve highly selective tumor-targeting ability *in vivo*, showing that H2a-4T@Cetuximab complex was potential for tumor theranostics. The *in vivo* distribution of H2a-4T@Cetuximab showed high *ex vivo* fluorescence signals in the liver and kidneys in mice at 24 hours p.i., demonstrating that the complex was mainly cleared through two pathways, including hepatobiliary and renal clearance pathways, especially by hepatobiliary clearance pathways owing to the large molecular weight of proteins (Figure 13F, G).

Additionally, programmed cell death ligand-1 (PD-L1) is overexpressed in many tumors. Plentiful evidence indicates that PD-L1 against tumor antigen-specific T cells forms the predominant mechanism of immune tolerance 199. Based on the above considerations, a probe (anti-PD-L1-BGP6) was synthesized with the covalent combination of the PD-L1 mAb-based on IR-BGP6 for NIR-II molecular imaging (Figure 13H) 135. Compared to the nonspecific NIR-II contrast agent, a significantly strong NIR-II fluorescence signal was accumulated in anti-PD-L1-BGP6 treated MC38 cells (Figure 13I). Significantly, after intravenous injection of anti-PD-L1-BGP6 into mice, the gradual assimilation of mAb *in vivo* made anti-PD-L1-BGP6 release IR-BGP6 with the ultrasmall HD of 3.5 nm, ensuring relatively rapid clearance that ~91% clearance by urine within the first 10 hours p.i. (Figure 13J). Importantly, the anti-PD-L1-BGP6 successfully realized efficient non-invasive *in vivo* molecular imaging with a tumor-to-normal tissue (T/NT) signal ratio of around 9.5. This work illuminates the mechanism of the renal-cleared NIR-II contrast agents and the advantage of *in vivo* NIR-II molecular imaging. Similarly, Dai group has reported a novel targeted peptide (CP) selectively combined with a biomarker CD133 of tumor stem cells 67. After being conjugated with a NIR-II fluorescent probe, the synthetic CP-IRT could be applied to *in vivo* NIR-II imaging. The CP-IRT probe has been confirmed to efficiently target the tumor in mice with a high T/NT (over 8). The core molecule IRT with D-A-D and hydrophilic side groups exhibited a QY of ~ 1.5% with a NIR-II fluorescence emission (peak:1050 nm). Remarkably, the CP-IRT size detected by DLS was ~ 5 nm (within the renal clearance range), contributing to rapid renal clearance from the body (~ 87% clearance within 6 hours). Therefore, the CD133 targeting NIR-II fluorescent probe are potential for cancer diagnosis and therapy in the clinical transformation. Significantly, affibody is another concerned protein for targeted bioimaging *in vivo*. Dai group has reported that CH1055-affibody (anti-EGFR-affibody, MW:~7.97KDa) was confirmed with a good imaging effect in tumors, and the T /NT reached about 15 at 6 hours, higher than that of CH1055-PEG (T/NT: ~ 3) 56. It was the first time to use a tumor-targeting NIR-II fluorescent probe to guide surgical resection. The biodistribution study of CH1055-affibody showed the high accumulation in the kidney by *ex vivo* NIR-II imaging. Still, only a low level of CH1055-affibody was detected in urine within 24 hours p.i., suggesting that the renal clearance was relatively slow due to the reabsorption of small proteins and peptides in the proximal tubules of the kidney.

##### 3.2.2.3. NIR-II contrast agents modified with water-soluble polymers

**Figure 14:**
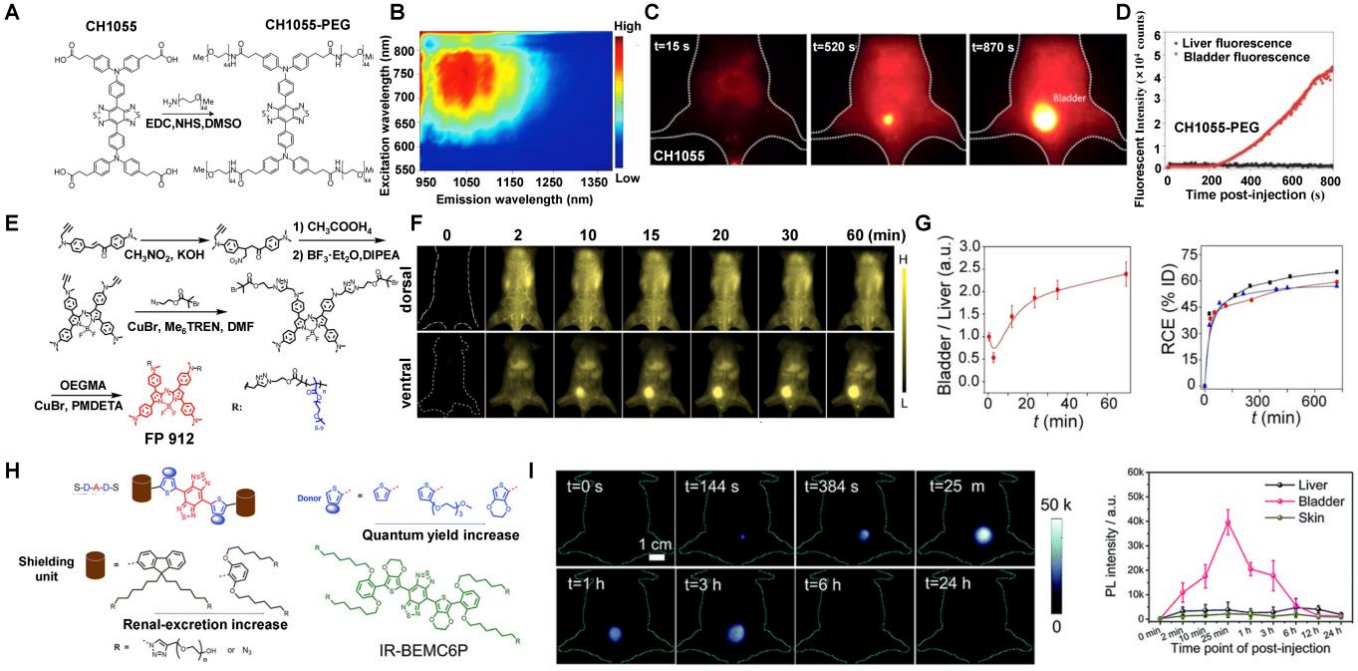
** Clearance of NIR-II contrast agents modified** with **water-soluble polymers.** (**A**) Chemical structure and synthesis of CH1055 and CH1055-PEG. (**B**) PL excitation spectra of CH1055-PEG. (**C**) Selected time points of CH1055-PEG from NIR-II imaging in a mouse with the supine position. (**D**) The representative fluorescent signal intensity of CH1055-PEG in both the liver and bladder regions (reproduced from ref. 56 with permission from Springer Nature). (**E**) Synthetic route of FBP 912. (**F**) *In vivo* bioimaging of FBP 912 in Balb/c mice with the dorsal or ventral position. (**G**) The ratios of bladder-to-liver intensity and renal clearance efficiency (RCE) as a function of time p.i. of FBP 912 in living mice (reproduced from ref. 136 with permission from Wiley-VCH Verlag GmbH). (**H**) Design the bright renal-clearance contrast agent IR-BEMC6P with shielding and donor group optimizations. (**I**) NIR-II imaging of the IR-BEMC6P in the mouse indicated high fluorescence signals in the bladder at different time points after intravenous injection (reproduced from ref. 66 with permission from Royal Society of Chemistry).

Most emitting cores of NIR-II contrast agents are a hydrophobic conjugated molecular skeleton, which requires external water-soluble chemical groups to gain water solubility. Based on the D-A-D NIR-II fluorescent materials study, Dai group has developed the NIR-II organic fluorescent probe CH1055, the first generation of D-A-D small molecule, for *in vivo* bioimaging (Figure 14A, B) 56. PEGylation has many advantages of long-term stability, circulation, and protection against the detection and degradation of the immune system 200. Herein, the PEGylated CH1055 was applied for *in vivo* NIR-II imaging with a peak fluorescent emission at 1055 nm. Of note, the HD of CH1055 was only about 3 nm with a molecular mass of 8.9 kDa, less than the renal filtration threshold. The pharmacokinetics of CH1055-PEG indicated rapid renal clearance with ~90% elimination *via* the renal system within 24 hours after intravenous injection (Figure 14C, D). Recently, Dai group also synthesized a series of D-A-D type fluorescent molecules by modifying the side chain of electron donor D, which greatly improved the fluorescence brightness of this molecule in the aqueous phase. For instance, the QY of IR-E1, IR-FEP, IR-FGP, and IR-FTAP could reach 0.7%, 2.0%, 1.9%, and 5.3%, respectively 56, 201. The average molecular weight of IR-E1 in water is about 4.5 kDa and its HD was about 3.6 nm. Nevertheless, IR-E1 has the advantages of good biocompatibility and renal clearance, but its QY was still low. While IR-FEP, IR-FGP, and IR-FTAP were slightly higher QY than IR-E1, they could not be quickly cleared from the body, leading to the toxicity increases of the material 201-203. Regarding the shortcomings mentioned above, Zhang group has developed a series of renal-clearable azaBODIPY brush NIR-II macromolecular fluorescence (named FBP) (Figure 14E) with an ultrasmall diameter of 4 nm in aqueous solution 136. What's more, FBP 912 exhibited the highest NIR-II brightness among all the FBP contrast agents and has a high renal clearance efficiency of ~ 65 % clearance *via* the renal system within 12 hours owing to the high water-solubility feature and the smaller size than glomerular filtration threshold (Figure 14F, G). Furthermore, FBP912 with prolonged circulation time (t^1/2^ ~ 6.1 hours) and rapid renal clearance (t^1/2^ < 2 h) contributed to actively targeted imaging in 4T1 tumor and renal cell carcinoma.

Importantly, Chen group has reported a novel NIR-II contrast agent named IR-BEMC6P (Figure 14H) based on the S-D-A-D-S structure optimization and structural characterization 66. Briefly, a small size less than the renal threshold is vital in providing renal clearance ability. Nevertheless, certain experimental results showed that size is not the only factor for renal clearance. After intravenously injecting IR-BEMC6P into CD-1 mice, the clearance of IR-BEMC6P was observed with 70% ID/g at 3 minutes p.i. in the blood circulation and decreased to <1% ID/g at 12 hours. The strong fluorescence signals of IR-BEMC6P in the bladder suggested a rapid renal clearance pathway (Figure 14I). Notably, the screened dialkoxy substituted benzene shielding groups as a critical factor for the IR-BEMC6P with renal clearance ability. Besides, when incubated these contrast agents with macrophages, IR-FEP and IR-FGP showed a much higher macrophage uptake (73.6% for IR-FGP, 99.4% for IR-FEP) in comparison with rapid clearance contrast agents (20.0% for IR-12N3, 32.4% for IR-BEMC6P), implying that long liver-uptake contrast agents were more easily internalized by macrophages. Meanwhile, surface chemistry also influenced renal clearance behaviors. By changing the azide group of IR-BEMC6P to amine and carboxyl groups, the fast renal clearance contrast agents could turn into high liver-uptake contrast agents. Therefore, it can be concluded that renal-clearance NIR-II contrast agents with the characteristics of small size, near-neutral chemical groups, rapid disaggregation with proteins, and low endocytosis by macrophages.

##### 3.2.2.4.NIR-II contrast agents modified by water-soluble ionization

**Figure 15:**
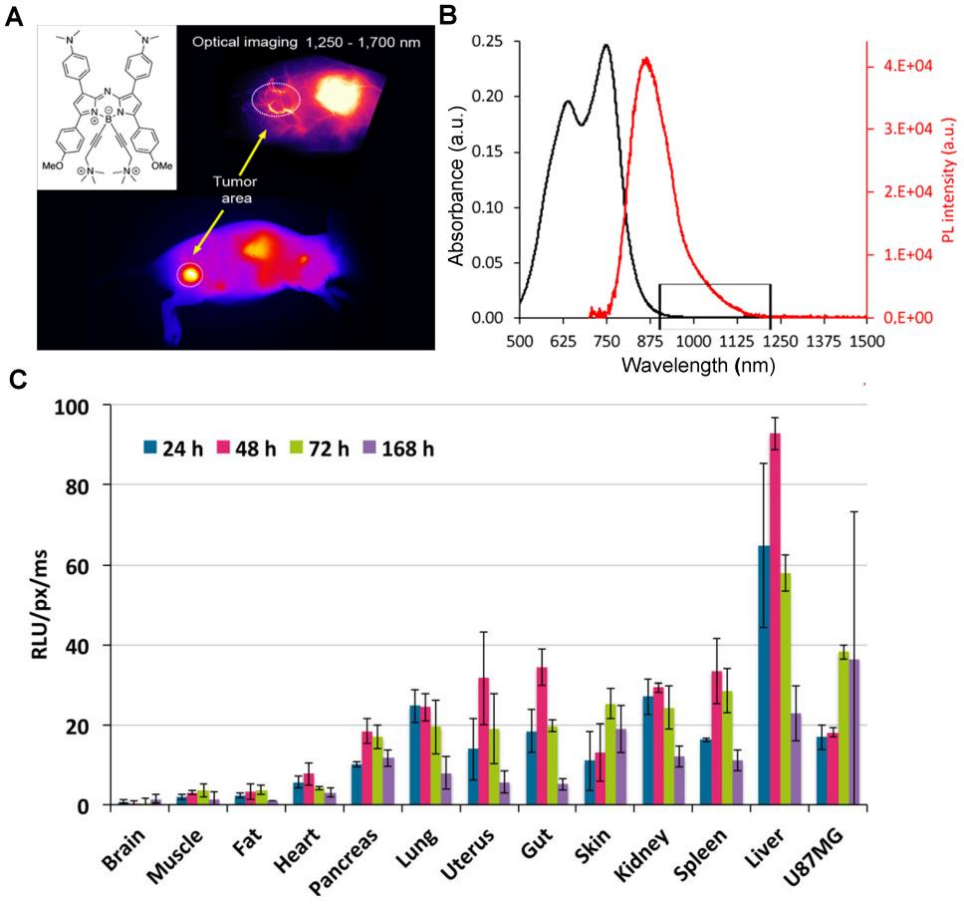
** Clearance of a NIR-II contrast agent modified by ammonium ionization.** (**A**) The structure and application of SWIR-WAZABY-01. (**B**) Absorption and PL emission range of SWIR-WAZABY-01. (**C**) The distributions were observed from 24 to 168 hours p.i. (reproduced from ref. 137 with permission from American Chemical Society).

According to their chemical structures, there are mainly two types of NIR-II molecular contrast agents emitting over 1000 nm, including donor-acceptor-donor (D-A-D) type contrast agents and polymethine contrast agents (D-π-A) type 2, 60, 66, 97, 204. In addition to the above of water-soluble matrix coating and water-soluble group modification, the ionic functionalization pathway (*e.g.,* sulfonic, phosphoric, and ammonium) of traditional small molecule contrast agents (*e.g.,* Polymethine, Cyanine, BODIPY, Rhodamine) is also an effective way for small hydrophobic fluorophores to achieve water-soluble property. Sancey group has reported a water-soluble ionization of NIR-II molecular fluorophore (SWIR-WAZABY-01) for NIR-II imaging with an obvious emission in the NIR-II window (Figure 15A, B) 137. After the intravenous injection of SWIR-WAZABY-01 into the U87MG tumor-bearing mice, the *in vivo* NIR-II imaging was monitored on day 7. The plasma half-life of SWIR-WAZABY-01 in mice showed that SWIR-WAZABY-01 presented 1.2 minutes for free circulating SWIR-WAZABY-01 and 55.5 minutes for SWIR-WAZABY-01 linked to plasma components* in vivo*. Inspiringly, it could rapidly be accumulated in tumors, reaching the maximum accumulation of NIR-II fluorescence imaging in the tumor sites at 72 hours p.i., then gradually be cleared from other organs, especially from the liver (Figure 15C).

**Figure 16:**
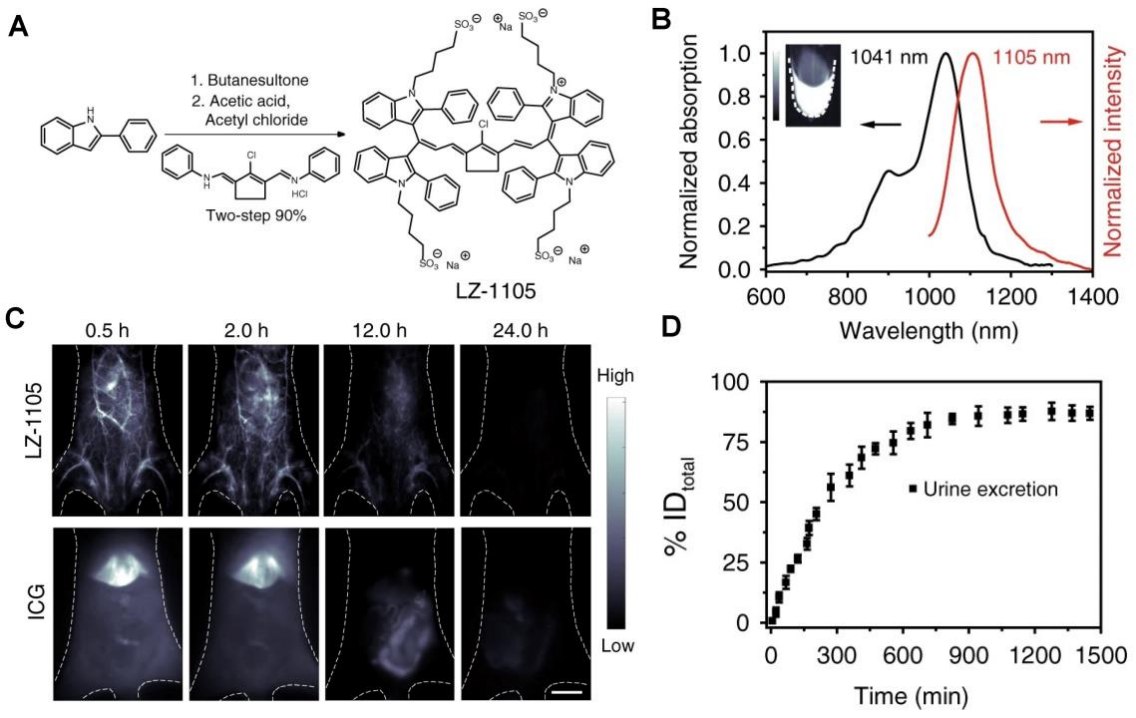
** Clearance of NIR-II contrast agents modified by sulfonic ionization.** (**A**) Synthetic route of LZ-1105. (**B**) Normalized absorption and fluorescence intensity of LZ-1105 in PBS. (**C**) NIR-II bioimaging *in vivo*. (**D**) Cumulative urine clearance curve of LZ-1105 in mice within 24 hours p.i. (reproduced from ref. [138]with permission from Springer Nature).

Besides, Zhang group has reported a water-soluble ionization of NIR-II molecular fluorescence LZ-1105 with long-term NIR-II imaging for dynamic vascular structural changes* in vivo* (Figure 16A) 138. LZ-1105, with a small molecular mass of 1.5 kDa, showed a peak fluorescent emission at 1105 nm (Figure 16B) with high resolution for non-invasive imaging for cerebral vasculatures of the mouse (Figure 16C). Significantly, the half-life of LZ-1105 was over 3 hours in blood circulation, providing a long imaging window to monitor the dynamic vascular structure changes. It was worth nothing that the clearance pathways of LZ-1105 and ICG were significantly different. ICG can be rapidly uptaken by the liver within 30 minutes, maintained for 6 hours, then cleared by hepatobiliary clearance, whereas only a weak fluorescence signal of LZ-1105 in the urine. Surprisingly, when mixed the urinated LZ1105 with blood, the signal in urine was enhanced up to 11.3-fold, identifying the LZ-1105 molecule in urine was in a state of free. After 24 hours of intravenous injection, about 86% of the injected dose (% ID) of LZ-1105 was observed in urine (Figure 16D), suggesting that LZ-1105 was cleared through the renal system. Herein, LZ-1105 opens a new way to evaluate the dysfunction of mice vessels with long excitation and emission wavelength, long-term blood circulation properties, and renal clearance.

##### 3.2.2.5. Water-soluble metallo-organic compound

**Figure 17:**
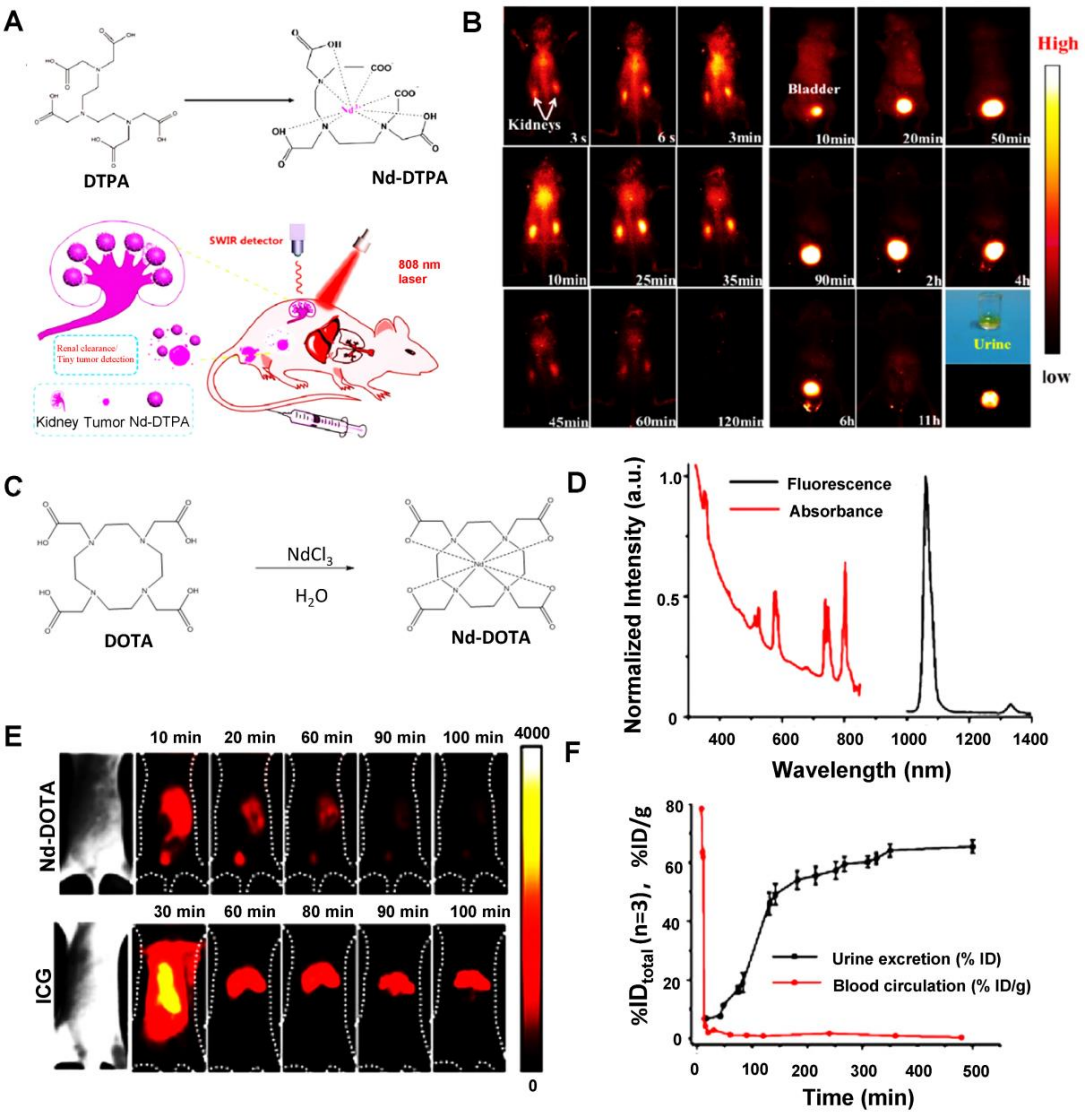
** Clearance of RENPs-based water-soluble metallo-organic compound.** (**A**) Schematic structure illustration of the DTPA and Nd-DTPA, along with the synthesis process of Nd-DTPA and bioapplication process in the NIR-II window under the 808 nm excitation. (**B**) *In vivo* NIR-II imaging of kidney signals of a mouse in the supine position (reproduced from ref. 139 with permission from Elsevier). (**C**). Chemical structure and the one-step synthesis of Nd-DOTA. (**D**) Absorbance and stimulated emission spectra of Nd-DOTA. (**E**). Selected time points from video-rate NIR-II imaging (850 nm and 1000 nm long-pass filter for ICG and Nd-DOTA, respectively) of a mouse in the supine position after an intravenous injection of Nd-DOTA (top) and ICG (down) showing disparate liver and bladder fluorescent signals (n= 3 mice) (**F**) Cumulative urine clearance curve of Nd-DOTA (% ID) as well as blood circulation (% ID/g) time points fitted with an exponential decay obtained during the 9 hours p.i. (reproduced from ref. 140 with permission from American Chemical Society).

As a lanthanide element, Neodymium (Nd), with unique electron structures, can absorb the light of 808 nm and emit NIR-II fluorescence at 1065 nm (^4^I_11/2_) and 1385 nm (^4^I_13/2_) 205. Owing to the advantages of narrow band emission, long transmission life, favorable photostability, deep penetration, and non-toxicity, Nd is a promising candidate for NIR-II fluorescence imaging, and various Nd chelate and Nd-contained nanoparticles have been designed for NIR-II fluorescence imaging 206, 207. Recently, Hao group has developed a new NIR-II emitting probe that based on the Nd-diethylene triamine pentacetate acid (DTPA) complex. The Nd-DTPA consisted of the chelation of the Nd^3+^and DTPA with molecular weight less than 40 kDa (Figure 17A). The NIR-II bioimaging of Nd-DTPA in Kunming mice was detected with the NIR-II emission centered at 1330 nm 139. As presented in Figure 17B, significant fluorescence signals in the kidneys were observed within a few seconds and gradually enhanced after 25 minutes of injection. A strong signal was also observed in the bladder within 10 minutes and fully cleared from the bladder within 11 hours, suggesting that the Nd-DTPA probe has rapid clearance from the kidney to the bladder for its small molecular weight. Furthermore, Nd-DTPA was firstly verified with optical-guided tiny tumor (down to ~ 3 mm) detection. These studies provide the potential for designing a new multi-modal small molecular probe for early tumor diagnosis and NIR-II imaging. Similarly, Zhang group has reported a rapidly cleared NIR-II lanthanide complex Nd-DOTA (Figure 17C) 140. Nd(III) organic complexes possess typical four energy level structures, a wide absorption band (400-900 nm), and emission at about 1057 nm in the NIR region (Figure 17D). The NIR imaging quality of Nd-DOTA, with good photostability and deep tissue penetration (7 mm), was better than that of clinically approved ICG (Figure 17E). The pharmacokinetics were evaluated by intravenously injecting Nd-DOTA into the mice and collecting the urine and blood over 9 hours. The half-life of Nd-DOTA in blood circulation was about 4 minutes. Additionally, Nd-DOTA with a molecular weight of 0.54 kDa, greatly below the renal filtration cutoff value (40 kDa), showing that a high accumulation in urine and kidneys was after 20 minutes p.i. and ~50% of the Nd-DOTA probe was cleared through the urine within 3 hours p.i. (Figure 17F). After intravenous injection of Nd-DOTA-FA, all the invisible small metastatic lesions are capable of being identified by NIR-II fluorescence bioimaging, directly speeding up the clinical transformation for this novel type of NIR-II probe in the future.

Small organic molecules with definite structures are an ideal material for NIR-II fluorescent probes because of their high biocompatibility, rapid clearance ability, and simple preparation methods. Hence, based on small organic molecules, the development of NIR-II contrast agents is still a hotpot 198. The design of NIR-II contrast agents largely depends on the combination of a strong electron donor (D) and acceptor (A), which can be easily modulated by various electron donors and acceptors 208. For the D-A-D structure, the spatial configuration of the strong electron donor groups on either side of the internal electron acceptor was to narrow the energy gap and push the final fluorescence emission wavelength into the NIR-II region. In order to get hydrophilicity, NIR-II molecular contrast agents are usually chemically modified with hydrophilic polymers or physically embedded in amphiphilic substances 209. Most of the molecular probes are smaller enough to pass through the glomerular capillary wall and enter the urine if they do not interact with serum proteins during circulation. In addition, with serum protein binding, NIR-II molecular contrast agents with enhanced hydrophobicity can only be cleared from the body *via* hepatobiliary pathways, including liver metabolism, bile, and fecal clearance 88. At present, some limitations of solubility and biocompatibility for organic contrast agents still exist. For example, small organic molecules are usually excreted faster than other kinds of NIR-II dyes. Nevertheless, they will remain in the body for hours to days and have *in vivo* toxicity to some extent, so it is necessary to study the excretion ability of contrast agents for clinical transformation.

## 4. Conclusion and outlook

NIR-II fluorescence imaging exhibit unique advantages of higher temporal and spatial resolution, which has splendid potential to achieve the high-precision, multi-dimensional, and cross-scale technical requirements in real-time investigations of biological processes. The favorable NIR-II contrast agents are vital in NIR-II imaging technology. To date, a growing number of NIR-II contrast agents, such as inorganic contrast agents, conjugated polymers, and small organic molecules, have been successfully applied to *in vivo* imaging and surgical navigation treatment. However, most NIR-II contrast agents, such as the large size of inorganic and polymer-based contrast agents, require days or even months to be entirely cleared from the body, causing toxic and side effects on the liver, spleen, and other organs, thus restraining the clinical transformation for NIR-II imaging technology. The ideal NIR-II contrast agents for clinical translation demands good optical properties and biocompatibility. Therefore, it is critical to understand the pharmacokinetic properties of NIR-II contrast agents *in vivo,* and subsequently optimize clearance pathways of NIR-II contrast agents to minimize toxicity by decreasing the duration of organ retention. The best NIR-II contrast agents can be designed by combining the *in vivo* biological effects with different clearance mechanisms. At present, there are three pathways to eliminate NIR-II contrast agents *in vivo* imaging, namely, MPS clearance (months to years), hepatobiliary clearance (hours to days), and renal clearance (minutes to hours) pathway. For the renal clearance pathway, in addition to the small size (< 6 nm) of foreign matters that can pass through the filtration barrier, another extremely important way of clearance is the phagocytose of renal mesangial cells. It is mainly for the large-sized or negatively charged materials, which can only be filtered through the fenestra of endothelial cells to enter the mesangial area. The premise of the hepatobiliary clearance pathway is that foreign objects can enter the perisinusoidal space from the hepatic sinusoid and be absorbed by hepatocytes. The hepatic sinusoidal window is 100~150 nm 210, only smaller foreign matters (< 100 nm) can pass through the hepatobiliary clearance pathway. Moreover, due to the negative charge on the surface of the hepatocytes, the positively charged intrusions can promote hepatobiliary clearance. In contrast, negatively charged intrusions can reduce the uptake of hepatocytes. MPS includes circulating monocytes, Kupffer cells, spleen red pulp, marginal zone macrophages, bone marrow macrophages, and sinusoid endothelial cells, among which the most representative cells are the Kupffer cells in the liver. Nanoparticles with a positive charge and slightly larger size (> 0.5 μm) 211 or increased HD diameter due to serum albumin binding formed protein corona.These intrusions cannot be cleared by renal or hepatobiliary pathway, they are mainly eliminated through MPS pathway with macrophage internalization and decomposition.

In view of these pathways, it can be inferred that renal clearance is the preferred pathway for NIR-II contrast agents due to rarely involving cellular internalization/metabolism, which can effectively reduce biological toxicity caused by the exposure of contrast agents to the body. To extend the applications of NIR-II contrast agents from their current preclinical stage to clinical translatability in diagnosis and treatment, numerous efforts have been devoted in the development of NIR-II contrast agents to gain favorable pharmacokinetics with body clearance performance, and significant progress has been made on nano-based and molecular-based of NIR-II contrast agents 56, 66. Nevertheless, most NIR-II contrast agents are still in their infancy. Although there are limitations in clinical transformation, there are still promising prospects for further improvement in this field. We envisage the main concerns regarding the engineering strategies of NIR-II contrast agents and provide some advice and prospects for further development of clearable and biocompatible NIR-II contrast agents.

First, the successful experience of traditional Vis-NIR contrast agents with renal clearance characteristics can be used as a reference in developing NIR-II contrast agents 75, 203. Rationally structured engineering strategies and surface modifications dedicated to reducing the adsorption of NIR-II contrast agents to visceral and biological tissues. By increasing their circulation in the cardiovascular system and regulating the diameter size lower than the renal filtration threshold (6 nm), NIR-II contrast agents achieved rapid renal clearance performance. The current studies have found that most existing NIR-II contrast agents remain in the liver and spleen, mainly by binding serum proteins (opsonin) *via* MPS. Therefore, purposeful chemical modification increases water solubility of NIR-II contrast agents and tunes amphoteric charge or neutral charge, or surface coating with biomimetic materials, which can effectively decrease liver and spleen accumulation, prolong the half-life of blood circulation and promote renal clearance of NIR-II contrast agents.

Second, the precision engineering strategy of the NIR-II contrast agents is an effective approach to accurately regulating their clearance properties 47, 74, 212, 213. Most existing nano-based NIR-II contrast agents are faced with drawbacks of morphology (size, shape) and surface characteristics (a charge, hydrophilicity, targeting) prone to dynamic change in the complex living body, resulting in unstable pharmacokinetic behaviors. The preparation of nano-based NIR-II contrast agents by physical entrapment holds the possibility of leakage. The micellar NIR-II contrast agents formed by self-assembly through amphiphilic interaction may alter after dilution* in vivo*. Preparing precise engineering NIR-II contrast agents with stable characteristics can immobilize clearance performance. Moreover, developing small molecular NIR-II contrast agents with controlled and stable physicochemical properties is also a considerable precision engineering strategy. Except for the clearance of the monodispersed NIR-II materials, inter-particle engineering may also enhance clearance 214. Using this novel strategy increased the accumulation of contrast agents at the tumor and effectively prolonged the retention time. NIR light-regulated disassembly would decrease the imaging background, accelerate the clearance rate of contrast agents, and reduce potential long-term biotoxicity.

Third, in view of another perspective, the purpose of developing clearance NIR-II contrast agents is to achieve biological safety, and other forms of administration (*e.g.,* oral, inhalation, spray coating) make it potential to address the long-term toxicity of some existing NIR-II contrast agents 71, 215, 216. According to the characteristics of the lesion sites, non-intravenous administration can avoid the possibility of imaging agents adsorbed by visceral organs in the blood circulation process. For example, *in vivo* imaging of intestinal flora and genital tract flora can be performed by the drench administration, and the spray coating administration can be used for lesions surgical navigation treatment.

Last but not least, obtaining biocompatibility NIR-II contrast agents from the nature of the biological source is a shortcut to breaking through biological safety 217-220 following the rule that “taken from nature, used in nature,” which can be artfully avoided the clearance pathways barriers that faced by the xenobiotics, artificial NIR-II contrast agents.

## Figures and Tables

**Table 1:** Clearance of inorganic NIR-II contrast agents

Materials type	Imaging agent	Excitation/Emission (nm)	Shape	Zeta (mV)	Size (nm)/MW (kDa)	Clearance pathway	Clearance duration	Ref.
SWNTs	DSPE-mPEG functionalized SWNTs	808/900-1500	Tube	-	-	MPS clearance	-	114
	LV SWNTs	808/1000-1800	Tube	-	Diameter: 0.96-1.24	MPS clearance	Over 7 days	115
Quantum dots	6PEG-Ag_2_S QDs	808/1100-1350	Sphere	-	DLS: 26.8 TEM: 5.8	Hepatobiliary clearance	Within 7 days	116
	Ag_2_Se QDs PEG	808/1100-1500	Sphere	-13.4	DLS: 29.6± 5.1 TEM: 5.0±0.8	Ag: hepatobiliary and renal clearance	Within 28 days. Se:un-excretion	117
	Ag_2_Te QDs	808/900-1400	Sphere	-4.6	DLS: 4.3 TEM: 3.0	Hepatobiliary and renal clearance	-	118
Rare earth materials	α-ErNPs	908/1400-1700	Crystal	-	DLS: 35.5	90% hepatobiliary clearance	Within 14 days	119
	RENPs@Lips	808/1000-1400	Sphere	~-25~-30	DLS: 105 ± 5.2 TEM: 72.2 ± 1.6	98% hepatobiliary clearance	Within 72 hours	120
	DCNP@GSH	808/1025-1100	Sphere	0.667	DCNP@GSH: DLS: 6	~80% renal and hepatobiliary clearance	Within 14 days	121
	RENPs@DSPE-mPEG	808/1000-1100, 1300-1400	Sphere	~-30~-35	DLS: 126.7 ± 1.5 TEM: 70.0 ± 2.6	MPS clearance	Within14 days	122
	Ir-B-TiO_2_@CCM	1064/500-750	Sphere	-4.38 ± 2.99	DLS: 389.15 ± 4.04	Renal (main) and hepatobiliary clearance	-	123
Other inorganic materials	Liquid metal@SiO_2_-RGD	1064/-	Sphere	~20	DLS: 236.43	Renal clearance: 55.7% of Ga	Within 7 days	124
	Au25 cluster	808/900-1400	Crystal	-	DLS: 3.2 TEM: <2	86.6% renal clearance, 2.2% hepatobiliary clearance	Within 48 hours	125
	CDs	808/900-1200	Sphere	-4.9	DLS: 4-12 TEM: 6	65% renal clearance	Within 6 hours	126

**Table 2:** Clearance of organic NIR-II contrast agents

Materials type	Imaging agent	Excitation/Emission (nm)	Shape	Zeta (mV)	Size (nm)/MW (kDa)	Clearance pathway	Clearance duration	Ref.
NIR-II contrast agents based conjugated polymers	PDFT1032	808/900-1400	Sphere	-	DLS/TEM: 68	Hepatobiliary and renal clearance.	-	127
	TTQ-2TC NPs	808/900-1400	Sphere	-	DLS: 140 TEM: 130	Hepatobiliary clearance.	-	128
	DPP-BTzTD Pdots	1064/760-860	Sphere	L-dots: -39 S-dots: -26	DLS/TEM: L-dots: 25 S-dots: 4	L-dots: hepatobiliary clearance. S-dots: hepatobiliary and renal clearance	Within 144 hours; Within 72 hours	129
NIR-II contrast agents encapsulated in amphiphilic matrixes	p-FE	808/900-1400	Sphere	-	DLS/TEM: 12	Hepatobiliary clearance	Within 3 days	35
	DTTB@PEG	808/900-1400	Sphere	-	DLS/TEM: 80	MPS clearance	-	130
	BPN-BBTD NPs	785/800-1300	Sphere	-	DLS/TEM: 37.1 ± 2.3	Hepatobiliary clearance	Within 33 days	131
	HLZ-BTED dots	808/900-1400	Sphere	-16.3	DLS: ~60 TEM: ~50	Hepatobiliary clearance	-	132
NIR-II contrast agents encapsulated in or modified with proteins	IR820-HSA	808/1000-1200	-	-	-	Hepatobiliary clearance	-	133
	H2a-4T@ Cetuximab	808/950-1500	-	-	-	Hepatobiliary and renal clearance	-	134
	Anti-PD-L1-BGP6	808/900-1400	-	-	IR-BGP6: DLS: 3.5	91% renal clearance	Within 10 hours	135
	CP-IRT	808/900-1400	-	-	DLS: ~5	87% renal clearance	Within 6 hours	67
	CH1055-affibody	808/900-1300	-	-	CH1055: MW: 0.97 Affibody: MW: ~7	Renal clearance	Over 24 hours	56
NIR-II contrast agents modified with the water-soluble polymer	CH1055/PEG	808/900-1300	-	-	DLS: 3 MW: 8.9	~90% renal clearance	Within 24 hours	56
	FBP912	808/900-1200	-	-	DLS: ~ 4	~65% excretion through the kidney	Within 12 hours	136
	IR-BEMC6P	808/900-1400	-	-	-	Renal clearance	Within 24 hours	66
NIR-II contrast agents modified by water-soluble ionization	SWIR-WAZABY-01	707/800-1500 in DMSO; 638/720-1200 in plasma.	-	-	-	Hepatobiliary clearance	_	137
	LZ-1105	1064/1000-1400	-	-	-	86% renal clearance	Within 24 hours	138
Water-soluble metallo-organic compound	Nd-DTPA	808/1000-1200, 1300-1400	-	-	MW: < 40	Renal clearance	Within 11 hours	139
	Nd-DOTA	808/1000-1200, 1300-1400	-	-	MW: 0.54	~50% renal clearance	Within 3 hours	140
